# Building adaptive capacity of US Gulf Coast city practitioners to extreme weather events

**DOI:** 10.1093/pnasnexus/pgag330

**Published:** 2026-09-28

**Authors:** Gabrielle Wong-Parodi, Natalie Herbert, Sarah J Fendrich, Alexander B Basaraba, Simone J Domingue, Erica A Goto, Patty Hernandez, Teal Harrison, Kripa Jagannathan, Jenna L Jorns, Anima Scott Kalafatis, Lisa Maillard, Alexander Petersen, Mark Shafer, Joshua J Wimhurst, Derek Van Berkel, Maria Carmen Lemos

**Affiliations:** Department of Earth System Science, Stanford University, Stanford, CA 94305, USA; Woods Institute for the Environment, Stanford University, Stanford, CA 94305, USA; Department of Earth System Science, Stanford University, Stanford, CA 94305, USA; Emmett Interdisciplinary Program in Environment and Resources, Stanford University, Stanford, CA 94305, USA; Emmett Interdisciplinary Program in Environment and Resources, Stanford University, Stanford, CA 94305, USA; ByWater Institute, Tulane University, New Orleans, LA 70118, USA; International Resilience Lab in Arizona Institute for Resilience, University of Arizona, Tucson, AZ 85721, USA; Headwaters Economics, Bozeman, MT 59715, USA; Adaptation International, Austin, TX 78704, USA; Earth and Environmental Sciences Area, Lawrence Berkeley National Laboratory, Berkeley, CA 94720, USA; School for Environment and Sustainability, University of Michigan, Ann Arbor, MI 48109, USA; Northwest Climate Adaptation Science Center, University of Washington, Seattle, WA 98195, USA; School for Environment and Sustainability, University of Michigan, Ann Arbor, MI 48109, USA; Adaptation International, Austin, TX 78704, USA; Department of Geography and Environmental Sustainability, University of Oklahoma, Norman, OK 73019, USA; Department of Geography and Environmental Sustainability, University of Oklahoma, Norman, OK 73019, USA; School for Environment and Sustainability, University of Michigan, Ann Arbor, MI 48109, USA; School for Environment and Sustainability, University of Michigan, Ann Arbor, MI 48109, USA

**Keywords:** extreme weather events, social experiment, longitudinal, climate adaptation, cities

## Abstract

Building city practitioners’ capacity to prepare for and respond to climate change-driven events can increase coastal cities’ resilience. We conducted a first-of-its-kind mixed methods research project from 2021 to 2023 with 46 US Gulf Coast small and midsize cities to measure the effectiveness of an adaptive capacity-building intervention: a stormwater planning tool called FloodWise Communities (FWC) deployed through the engagement between researchers and practitioners in each city. City practitioners were surveyed preintervention and postintervention between September 2021 and December 2022. Interviews were then conducted through July 2023. We found (i) FWC positively relates to perceptions of actionable knowledge and a change in adaptation intentions; (ii) city practitioners’ adaptation-relevant roles and responsibilities positively related to their perceptions of actionable knowledge and adaptation intentions, with implications for their agency to build adaptive capacity; and (iii) anticipated and unanticipated uses of the FWC tool influenced city practitioners’ perceptions of their adaptive capacity.

Significance statement In a first-of-its-kind mixed methods research project conducted from 2021 to 2023 with 46 US Gulf Coast small and midsize cities, we found that a stormwater planning tool called FloodWise Communities helped build city practitioners’ capacity to prepare for and respond to climate change-driven events.

## Introduction

Approximately 2.9 billion people reside in coastal areas (1) and are increasingly at-risk from global environmental change. Many coastal cities have aging or informal stormwater infrastructure (2, 3) that is ill-equipped to handle intensifying climate change impacts. One such place is the US Gulf Coast, which has the highest population growth of any other coastal region in the United States, increasing by 26.1% from 2000 through 2017 (4). Rapid urbanization and growth in Gulf Coast cities, suburbs, and towns is expected to continue unabated in the near future driven by in-migration, rural development, and urban sprawl (5–7). The region is experiencing particularly rapid and severe impacts of climate change-driven events (8, 9) due to its location, topography, and socioeconomic conditions (10). These events include chronic stressors, such as sea level rise (11) and associated saltwater intrusion (12), and acute shocks, such as tropical cyclones (hurricanes) that bring heavy precipitation and flooding (13, 14). Since 1980, the US Gulf Coast has experienced, on average, $5.2 billion in damages from weather and climate disaster events per year (15), comprising more than 64% of all disasters in the United States in a given year. According to the Federal Emergency Management Agency National Risk Index, as of December 2024, estimated annual loss from all weather and climate events in coastal counties in the Gulf region is ∼$8.97 billion, with losses of $7.55 billion in buildings, $1.36 billion in loss of life (about 117 fatalities), and $50 million in agriculture (16). This places the region in the 93rd percentile in average annual loss rate in the United States (16). Gulf Coast cities, like others around the world, must manage increasing hazards for residents with infrastructure not built for such extreme conditions. Adding to the challenge are constraints on the capacity of small cities for assessing, planning, and implementing adaptation action. In the US Gulf Coast, smaller cities and surrounding areas—particularly those with higher proportions of lower-income communities and underrepresented racial and ethnic groups—often do not have the capacity to plan for and use funds to adapt and evaluate their stormwater infrastructure to the impacts of a changing climate (17).

In this article, we describe a first-of-its-kind mixed methods research project using a social experiment design with 200 practitioners from 46 Gulf Coast small and midsize cities (populations <250,000) located within 100 miles from the coast. Specifically, we tested if an adaptive capacity-building intervention (deploying a decision-support tool [DST] through practitioner and researcher engagement) increased city practitioners’ understanding of new ideas and information (“learning”) and the belief they have the power to act on this knowledge (“agency”). Here, we build upon extant literature that conceptualizes adaptive capacity primarily in relational terms, especially learning. In several influential adaptive capacity frameworks, learning is not only its own indicator of adaptive capacity but also contributes to other indicators (18–21). Because of their variable roles and responsibilities, not all practitioners have the same ability to incorporate new ideas and information into their work, suggesting that practitioners have varying levels of agency. Thus, our social experiment is conducted in the context of city practitioners who will likely make consequential decisions from what they have learned such as those regarding how to increase the resilience of their cities’ stormwater infrastructure (22).

Over the past 30 years, researchers have developed many approaches to define adaptive capacity (the ability to adjust to challenges) and how to build it (23). Early ideas suggested capacity grows when people have access and can mobilize different types of resources or “capitals” like money, education, social networks, cultural knowledge, natural resources, institutions, political power, and information (24, 25). More recent approaches look beyond capitals to focus on processes and relationships, such as being flexible, learning from experience, and agency (26–28). (See Supplementary information section below for more details).

While researchers increasingly agree on what determines adaptive capacity (23, 29), measuring it remains difficult (29–31). This is because adaptive capacity is unobservable until a disturbance happens that necessitates adaptation. Assessing adaptive capacity requires having a baseline against which to measure change (30). Researchers disagree on how to establish this baseline and track changes, especially in real-world situations (23, 30). One promising approach is to conduct social experiments testing whether capacity-building interventions actually affect practitioners’ real-world decisions (22). Our mixed-methods study addresses this gap by measuring city practitioners’ learning and agency before and after an adaptation capacity-building intervention.

The adaptive capacity-building intervention at the heart of this study is an online freely available adaptation planning DST called Floodwise Communities (FWC) (32). DSTs are one way that city practitioners may access high-quality scientific knowledge about climate change-driven hazards and adaptation solutions. Adaptation planning DSTs are typically developed to support decision-making involving computationally intensive analysis coupled with mapping of hazards to exposures (33). Yet simply having access to scientific knowledge may not drive city practitioners toward solutions to societal problems (34). Empirically, there is growing evidence that engagement between practitioners and researchers can make scientific knowledge, such as that presented through a DST, more actionable (19). Influential models of usable or actionable knowledge argue that: (i) knowledge that is *credible* and *legitimate* is more likely to be actionable (35); and (ii) knowledge that *fits* decision-making contexts, *interplays* positively with other kinds of knowledge already shaping decisions, and is *coproduced* is more likely to be usable in decision-contexts (35). Hence, city practitioner perceptions about the actionability of a DST may be more positive after engagement with researchers.

Many levels of government are responsible for adaptation decision-making. We use the term “cities” to encompass local governments operating stormwater systems at multiple levels such as cities, towns, parishes, villages, and counties. City practitioners participating in this study included those with a range of roles across administration, public works, engineering, planning, zoning, building management, environmental management, and emergency management. These different roles may have varying levels of agency to implement learning into adaptation.

For this mixed methods research project, we specially developed a web-based stormwater planning tool (35) building on the success of a spreadsheet-based tool coproduced by practitioners and researchers in the Great Lakes region; the web-based tool is the result of collaboration between climatologists, Great Lakes practitioners, adaptation planners, and web designers (for more information about the tool, please see Supplementary information). Teams of city practitioners were randomly assigned to different types of high-quality engagement with a team of scientists and extension experts to use the tool in their planning processes, either through in-person workshops, webinars, or self-guided tutorials available online, containing extensive video and written training materials (for more information about the engagement process, please see Supplementary information). In our study, we referred to the tool plus any of these high-quality engagements as FWC. Through FWC, participants learned about local climate hazards, performed vulnerability assessments, and developed potential adaptation actions specific to their stormwater infrastructure. This reflects the decision support needs and adaptive capacity building the participants reported needing preintervention. All city practitioners had personalized access to FWC to use at their convenience. Participants completed a preintervention survey on their (i) perceptions regarding anticipated use of FWC and (ii) intended adaptation actions. They also answered questions related to anticipated (iii) fit, (iv) legitimacy, (v) credibility, and (vi) interplay. After participants completed the FWC intervention and developed a city-specific plan to complete their vulnerability assessment and integrate results into next steps, they completed a postintervention survey on the same measures. A specific goal of the FWC intervention is to increase adaptive capacity learning in the context of stormwater management; while FWC may build adaptive capacity at different scales, our focus for this article is at the scale of individual city practitioners.

We first hypothesized that (H1) after the intervention, we will observe positive perceptions about the actionability—fit, legitimacy, credibility, interplay, and usefulness—of the FWC tool and a change in intended adaptation actions in either direction among city practitioners. We tested this hypothesis by having our 200 city practitioners complete surveys between September 2021 and December 2022 where they reflected on their own adaptive capacity, and reported on their perceptions of the FWC tool and intended adaptations before and after the intervention. The direction of adaptation intentions could stay the same, increase or decrease depending on how city practitioners interpret their vulnerability during the intervention. Cinner et al. (26) suggest that practitioners’ agency matters in terms of whether they can put adaptive capacity learning into practice. We translated the concept of agency into our measure of adaptation-relevant personnel (ARP), measured as the number of participants on a city team who scored highest on functions associating closely with adaptation planning and implementation (Fig. 1). We also hypothesized (H2) that city practitioners’ adaptation-relevant roles and responsibilities will relate to—strengthen or weaken—their perceptions about the actionability of the FWC tool and intended adaptation actions. Prior research suggests that the perceived relevance and use of climate information can vary by practitioners’ expertise, training, professional background, and organizational position (36, 37). Therefore, practitioners occupying different adaptation-relevant roles may evaluate and engage with DST differently (36, 37). Responses from the surveys were used to test H2.

**Figure 1:**
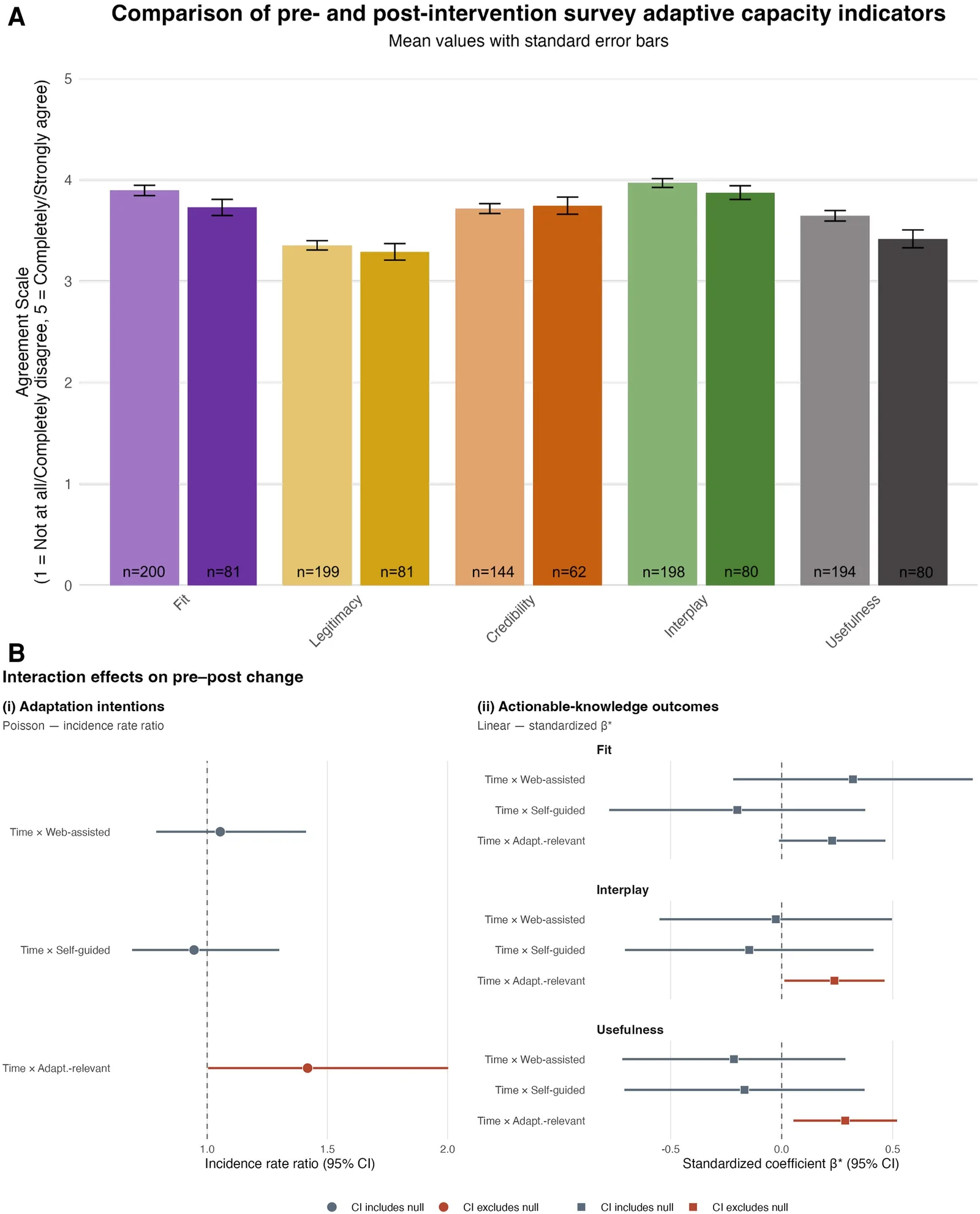
A) City-level availability of capitals for stormwater adaptation (*n* = 200, 46 cities) and B) city team coverage of roles relevant to adaptation decision-making. Tukey-style boxplots in which the vertical lines within each box represent the median value and box limits are IQR for each item. Lower whiskers include values greater than or equal to the first quartile minus 1.5×IQR; upper whiskers include values less than or equal to the third quartile plus 1.5×IQR. Points beyond whiskers represent outliers, which fall outside the defined 1.5×IQR in either direction from the box limits. Source: preintervention surveys (*N* = 200). A) Units of analysis are city teams. For each adaptation capital, values represent the proportion of participants within a city team who reported that capital was available in their community. For example, a median value of 50% for technical expertise and funding indicates that, in the median city, half of the city team reported access to those forms of capital. B) Units of analysis are city teams. Values represent the total number of personnel on a city team reporting this role (binary) and are not averaged by the number of practitioners on a city team. The gray box (top, separated by a dotted line) shows the distribution of total ARP per team. ARP were defined as participants who performed three or more of the following roles. Note that this figure only represents the participants in the FWC process (not all personnel that work at the city), and that we encouraged cross-department collaboration.

At the end of the intervention period of the study, we felt confident that we could assess city practitioners’ perception of the actionability of the FWC tool and adaptation intentions. However, we still did not understand how the intervention impacted their perceptions of their adaptive capacity given the diversity of decision-contexts and use of the FWC in practice. To explore this, we conducted in-depth follow-up interviews with 43 practitioners from May 2022 to August 2023.

Before reporting on our main findings, we present participants’ self-reported information on the capitals available in their cities (24, 25) to support stormwater infrastructure management or actions. Participants varied in proportion by the level of capitals they perceived as available or not in their cities (Fig. 1A). Notably, most participants reported that data about their stormwater system's vulnerability was not an available resource. However, participants reported they had other capitals available to them, including leadership support in their cities (approximately three-quarters of participants). Additional considerations of adaptive capacity are the varying functions city practitioners perform in their roles and how they may participate more or less regularly in actions related to adaptation decision-making and implementation (Fig. 1B), which are indicators of their agency (26, 27). We revisit this aspect of participants’ agency in the Results section below.

## Results

### Positive initial perceptions about FWC maintained postintervention, while intended adaptation actions decreased

We hypothesized (H1) that, after the intervention, we would observe positive perceptions about the actionability of the FWC tool and a change in intended adaptation actions in either direction among city practitioners. For example, increased knowledge could make practitioners increase their adaptation intentions (e.g. learning that existing gray infrastructure is more sensitive, increasing intentions to implement green infrastructure) or decrease them (e.g. learning that many components have high adaptive capacity, lowering system vulnerability). On balance, our findings largely support H1. Participants reported that their city intended to adopt six adaptation actions before the intervention (Table S2). After the intervention, intentions decreased to five on average. Of those adaptation intentions selected by the majority of participants (more than 50%), we observed a significant decrease for “gathering more information about hazards” (76% preintervention to 60% post, χ^2^ = 6.55, *P* = 0.011), “maintaining drainage ditches and pipes for higher run off” (76% preintervention to 61% post, χ^2^ = 6.05, *P* = 0.014), and “installing detention or retention ponds to control runoff” (67% preintervention to 48% post, χ^2^ = 8.57, *P* = 0.003). See Table S3 for more details. Indicators of actionable knowledge, however, mostly decreased during the study. Overall, participants perceived fit, legitimacy, credibility, interplay, and usefulness as moderately positive before the intervention and after (Fig. S5, Table S2).

### The right team and the right tools

We hypothesized (H2) that city practitioners’ adaptation-relevant roles and responsibilities would relate to—strengthen or weaken—their perceptions about the actionability of the FWC tool and intended adaptation actions. We found support for H2. Our analyses revealed that having a greater number of ARP in a participating city was associated with greater perceived tool fit preintervention (*β* = 0.289, 95% CI [0.039, 0.539], β* = 0.156, *z* = 2.278, *P* = 0.023, Table S5). Our analyses also revealed ARP were more likely to rate higher interplay (*β* = 0.375, 95% CI [0.003, 0.748], β* = 0.238, *z* = 1.986, *P* = 0.048, Table S8), usefulness (*β* = 0.559, 95% CI [0.095, 1.023], β* = 0.286, *z* = 2.372, *P* = 0.018, Table S9) and, importantly, adaptation intentions (incidence rate ratios [IRR] = 1.419, 95% CI [1.004, 2.004], *z* = 1.993, *P* = 0.047, Table S4) following intervention.

### Different kinds of FWC tool use in practice affects perceptions of adaptive capacity

Our results show that the intervention impacted the perceptions of the FWC tool and reported adaptation intentions of participating city practitioners. Yet given the complexity of the decision context and subsequent diversity of uses of the FWC tool in practice, we wanted to know more about *how* the intervention impacted adaptive capacity perceptions.

Through our interviews, we sought to better understand how participants describe using or not using what they learned from the FWC tool for adaptation actions more broadly. We also sought to explore whether their use or nonuse was associated with the mention and level (*high* vs. *low or no*) of specific actionable knowledge indicators measured in the study (i.e. fit, interplay, usefulness, credibility, and legitimacy). Through our inductive and deductive approach to coding of the interviews, we identified three different types of use. *Instrumental use* is defined as using the tool in stormwater management processes, adaptation planning and/or implementation. We also coded for two additional kinds of actions drawing from the literature of the intersection between social science and public policy (38–40). *Symbolic use* refers to confirmation of previously held beliefs or opinions, or learning that legitimates existing decision-making structure and plans. *Conceptual use* refers to learning that expands one's understanding of a problem—rather than implementable now (instrumental use), this learning is influential later. Scholars in the field of sustainability and climate action use this theoretical model of instrumental, symbolic, and conceptual use in their study of coproduced and community-engaged research processes (41).

Code occurrence in all interviews ranged from 6 to 36 references (Table 1). Fit was the most commonly coded actionable knowledge indicator in our interviews, particularly as it overlapped with descriptions of instrumental and conceptual use. A positive association between fit and instrumental use is exemplified by interviewee 24:

“I think it's very relevant especially with the fact that over the next year we'll be developing our climate action plan. Also, I’m the liaison for our Citizen Climate Advisory Committee as well, which dives into all these issues. So, it'll be a tool and information that I can relate back to them, and then they can also utilize and provide recommendations to our County Commissioners, which are our policymakers. So it should be a helpful tool for them as well.” (interviewee 24)

**Table 1:** Instrumental, conceptual, and symbolic use and nonuse of the FWC tool as related to indicators of actionable knowledge.

		Instrumental		Conceptual		Symbolic	
		Use	Nonuse	Use	Nonuse	Use	Nonuse
Fit	High	61	8	29	0	8	0
	Low or no	6	11	3	0	1	0
Interplay	High	12	1	2	0	4	0
	Low or no	0	1	0	0	0	0
Usability	High	2	0	0	0	0	0
	Low	0	1	1	0	0	0
Credibility	High	1	1	2	0	2	0
	Low or no	1	0	0	0	0	0
Legitimacy	High	2	2	3	0	0	0
	Low or no	0	2	0	0	0	0

In contrast, other interviewees did not use the tool despite reporting a high level of fit. These interviewees described the tool as being a good fit in terms of being a “good educational experience” (interviewee 37) and as a way to have “a good conversation…even if they [stormwater management decision-makers] don’t start actively using the tool” (interviewee 28).

On balance, we observed very little nonuse of the FWC tool, and no conceptual or symbolic nonuse as it overlapped with actionable knowledge indicators. One plausible explanation is that conceptual or symbolic nonuse are harder to see and articulate. For example, insistence about not learning anything (conceptual nonuse) or resisting opinion and/or belief change (symbolic nonuse) are unlikely to come up in the context of an interview. However, we did observe some instrumental nonuse, and much of that was explained with low fit: “the value was limited in my role currently” (interviewee 26); “…we don’t really have huge apartment complexes and things like that that would necessarily be quote unquote a ‘vulnerable group’” (interviewee 22). This makes sense for participants who joined the project with less adaptation-relevant roles (e.g. public safety, mayor's office) (22). Notably, usefulness, credibility, and legitimacy came up very infrequently in the interviews alongside comments about use. Credibility and legitimacy may not be top-of-mind when participants reported on tool use, or participants may feel disinclined to share candid views with researchers who had some hand in developing FWC.

One overarching theme that emerged from the interviews was the importance of relationships between practitioners within a city for building adaptive capacity. Being in the same room as others working on stormwater management but from different perspectives (42) and roles was connected to building adaptive capacity, which may not be measured on traditional indicators or on the timescale of the study. As stated by interviewees 3 and 4:

“We all–I hate to say we're compartmentalized– but each department has its own regulatory documents on how to handle flooding, infrastructure, stormwater, all those types of things. So Environment (department) regulates a portion, and Engineering (department) regulates a portion. And this was an opportunity for all of the departments to get in one room and kind of see what everybody else in the city is doing, what the other departments are doing, and maybe figure out how to integrate that into our overall planning where we help each other.” (interviewees 3 and 4)

## Discussion

Overall, we found that the intervention variably impacted city practitioners’ perceptions of their adaptive capacity. We found city practitioners’ intentions to implement adaptation actions went down over time, while most indicators of actionable knowledge remained robust after the intervention. Our interviews and extant literature suggest this could be because previously intended adaptations are more than sufficient (43), in conflict with another department, or are not implementable. Nevertheless, we did see an increase in intentions for “natural or green infrastructure projects” from 45% of participants preintervention to 57% post (χ^2^ = 3.02, *P* = 0.082), possibly reflecting the tool's emphasis on low-impact design or the availability of funding and the larger national discourse on nature-based solutions. This suggests that the intervention may have also helped city practitioners better understand the vulnerability of their stormwater systems and of their adaptive capacity, which may also contribute to building adaptive capacity as practitioners have a better sense of where to target their efforts. For example, one interviewee reflected on how the intervention shifted their focus: “…with the rainfall happening so much in smaller boroughs, it's made me think… we need to focus on smaller moments and make sure that when we do put a culvert in our drain ditch and… that it's able to take more water than just the average” (interviewee 6). In addition, web-based engagements using platforms such as Zoom or Skype (44) may help scale DSTs for adaptation planning.

### Limitations

While the experimental component of the study was originally designed as a randomized controlled trial, it contextually evolved into an “on-the-ground” intervention due to two severely disrupting events—the COVID-19 pandemic and the landing of 2022's Hurricane Ian, which both dramatically affected our participating cities and practitioners. On-the-ground interventions, in the tradition of societal experimentation approaches, test ideas, and innovations in urban settings (37). These experiments, rather than being strictly controlled, embrace the “messiness” of their own humanity (22). In our study, this humanity was critically shaped by the impact of the COVID-19 pandemic and Hurricane Ian, which significantly affected our ability to carry out the different stages of research (with preintervention and postintervention data collection and interviews). We began our study after the national pandemic emergency was declared on 2020 March 13, and as a result, recruitment started in December 2020. Despite many challenges affecting our targeted small cities, we were able to recruit 46 cities and strived to make accommodations as needed to maintain our commitment to ethical, responsible, and respectful research practices (22). Later, when Hurricane Ian made landfall as a category 4 storm in Florida on 2022 September 28 during postintervention survey data collection, we also faced substantial challenges. Because many of our participants in Florida were dealing with the hurricane and its aftermath, we elected to not pursue data collection in Florida, which contributed to a relatively low postintervention survey response rate (see Methods for more details). Two hundred participants completed the preintervention survey and 84 completed the postintervention survey.

Everything that our city teams were grappling with from COVID-19 to landfalling hurricanes illustrates the real-world challenges small cities with limited resources and low capacity face when trying to manage their stormwater systems in an era of a rapidly changing climate. This could have implications for who was able to engage fully, for example, those who are more or less familiar with stormwater systems or likely to make adaptation decisions. This could also have implications for survey completion rates. Indeed, our *t*-tests examining the association of key measures with survey completion status (those who completed preintervention and postintervention survey vs. those who completed preintervention only) found differences for baseline levels of tool fit (*t* = 2.07, *P* = 0.039), interplay (*t* = 2.45, *P* = 0.015), and credibility (*t* = 3.20, *P* = 0.002); there were no differences in completion status for intended adaptation actions, usefulness, ARP, or legitimacy. An ANOVA test of attrition by group found differences (F(2, 325) = 3.697, *P* = 0.026). An ANOVA test of differences in attrition by group suggests that the contrast driving this result is the difference in attrition between in-person (40% attrition) and self-guided (58% attrition) groups (difference = 0.185, *P* = 0.022). Despite attrition, we were able to measure differing adaptive capacity outcomes by engagement type *and* also what engagement means for adaptive capacity in our small cities more broadly through qualitative analysis. Interviews conducted with some of our small city practitioners provided insight into the changes we observe regarding engagement and adaptive capacity and are used here to understand our results.

### Future directions

To more fully understand and evaluate the intervention and the FWC tool for adaptive capacity, future research could continue to follow our 46 FWC cities as they further develop adaptation plans and implement stormwater management projects. Infrastructure plans take a long time to develop, fund, and implement (45), and adaptation plans are no different depending on the proposed project, program, or policy. Following FWC cities over time would be especially important given postintervention attrition in this study due to the effects of Hurricane Ian and likely other unobserved stressors to cities during this time.

Our findings also suggest that to build capacity in cities, it is necessary to have staff who make adaptation-relevant decisions, and for whom DSTs (33) like FWC are useful. Tools must fit and match practice, and not vice versa. All cities that participated in FWC volunteered to do so because they had an interest in the support offered by the tool; however, they were not equally positioned to apply the tool and the information it contained to their stormwater system planning. Fit—the degree to which the tool was aligned with practitioners’ responsibilities, decisions, and local planning context—emerged as an important driver of perceived actionability and use in our interviews. This aligns with prior work showing that climate information use depends not only on the usability of information itself but also on the context of use, including practitioners’ responsibilities, organizational capacity, decision-making processes, and the broader policy environment (36). Consistent with this, FWC was perceived as more actionable by practitioners whose roles and responsibilities were more related to climate adaptation and stormwater planning. However, not all communities had at least one team member whose roles and responsibilities aligned with their adaptation needs or with the tool. Most notable were the small number of practitioners who work with climate or socioeconomic data, communicate about climate change to other practitioners, or contribute to climate action plans. Limited institutional investment in adaptation-specific roles and responsibilities may be appropriate and adequate in the near term, but not if we consider the increasing risk of inundation for all cities by 2100.

As the need for advancing climate resilient infrastructure increases, climate-smart investments that enable small coastal cities to access resources and build capacity that leads to more effective and proactive adaptation (46) efforts are essential. Building capacity means investing in people as well as in critical infrastructure that supports societal wellbeing over the long-term under a rapidly changing climate. And addressing the disproportionate impacts of climate change on frontline communities (47)—those impacted first and worst by climate change—means investing in transformative climate adaptation rather than taking a status-quo, “business-as-usual,” or incremental approach (48). Alongside this is the need to better identify and develop those tools that best support the decisions that these city practitioners need to make—and can be usable by the practitioners in their existing roles—to proactively adapt infrastructure to a changing climate. Finally, our “on-the-ground” intervention illustrates the power of this kind of experimental approach for both scholarship and practice.

## Methods

### Procedure

The study design is a field experiment with 46 coastal cities between September 2021 and December 2022. Cities were recruited to join this project beginning in December 2020 through July 2021, and interested cities applied to join the project through an online system. Eligibility was determined based on a priori criteria for size (small-to-medium sized communities, with fewer than 250,000 residents), within 100 miles of the Gulf Coast (Alabama, Florida, Louisiana, Mississippi, or Texas), who manage their own stormwater system. Some otherwise eligible cities were not included in the experiment because they did not manage their own stormwater system or overlapped in decision-making for stormwater with another enrolled city. We identified and invited over 300 cities who fit our eligibility criteria, of which 60 agreed to participate. Five cities overlapped with another enrollee in terms of managing the same stormwater system (i.e. a city within a county or parish) and were not enrolled in the experimental study. (See Fig. S1 and Table S1 for more information about the location and description of the cities.) Cities were randomly assigned to one of three engagement groups using states as clusters to ensure geographic variability across groups. Three cities ended their participation immediately after random assignment to condition; two cities dropped from the study after scheduling engagement; and four cities did not complete required pretest surveys to participate in the experiment. More information about FWC and the intervention procedures can be found below (Intervention section).

Each community had 1–9 representatives who participated in the study. City teams were encouraged to identify participants in their city that span many roles relevant to stormwater systems and adaptation planning. City teams often included stormwater engineers, floodplain managers, public works directors, emergency planners, and communication departments.

Research from the field of actionable knowledge suggests that engagement results in greater perceived fit (e.g. appropriate for the decision context), legitimacy (e.g. authoritative and relevant), credibility (e.g. the partners involved are believable), and interplay (e.g. does the information or process fit in with existing decision-making practices?) (35) of information and planning. Before engaging with FWC, city team members completed a preintervention survey programmed and collected through Qualtrics beginning on 2021 September 21. Survey data included measures of (i) perceptions regarding anticipated use of FWC and (ii) intended adaptation actions. Participants also answered questions related to anticipated (iii) fit, (iv) legitimacy, (v) credibility, and (vi) interplay. A total of 200 participants completed at least some portion of the preintervention surveys, and 84 completed at least some portion of the postintervention surveys. Participating cities were required to complete all team members’ preintervention surveys before the FWC team shared the tool and associated materials. The FWC team asked participating cities to complete postintervention surveys only after cities had communicated that they had completed using the tool for their vulnerability assessment. Participants completed the postintervention survey on the same measures, also on Qualtrics. The postintervention survey was kept in the field until 2022 December 15 to allow participants sufficient time to complete the study. We elected to not follow-up with participants from 19 cities residing in Florida after 2022's devastating Hurricane Ian, as many were involved in postdisaster recovery. Table 2 shows the counts by community of the number of preintervention and postintervention surveys completed. Analyses were preregistered on the Open Science Framework (https://doi.org/10.17605/OSF.IO/RJAHY).

**Table 2:** Preintervention and postintervention data collection for 46 coastal cities by state.

Cities	Preintervention	Postintervention	Hurricane Ian no follow-up?
AL			
1	5	3	No
2	6	2	No
3	1	0	No
4	7	3	No
FL			
5	2	0	Yes
6	3	0	Yes
7	5	2	Yes
8	5	5	Yes
9	4	1	Yes
10	4	5	Yes
11	4	2	Yes
12	6	0	Yes
13	7	3	Yes
14	4	0	Yes
15	5	0	Yes
16	4	0	Yes
17	6	1	Yes
18	3	0	Yes
19	1	0	Yes
20	4	2	Yes
21	3	3	Yes
22	9	3	Yes
23	6	1	Yes
LA			
24	4	1	No
25	6	5	No
26	5	0	No
27	5	4	No
28	5	2	No
29	5	2	No
30	1	0	No
MS			
31	3	2	No
32	7	6	No
33	4	3	No
TX			
34	2	2	No
35	3	2	No
36	3	3	No
37	4	0	No
38	4	3	No
39	4	1	No
40	2	0	No
41	6	2	No
42	3	2	No
43	4	0	No
44	4	3	No
45	4	4	No
46	8	1	No

We conducted a power calculation for change in preintervention to postintervention survey responses, assuming an effect size of 0.2, power of 0.8, an alpha of 0.05, and 10 covariates, which suggests a total sample size of 245 people (or about 82 people per group). Ultimately, we aimed to recruit 60 cities for the experiment, and completed the experiment (delivered intervention with some data collection) with 46 total cities: 15 cities (59 individual pretests, 22 post-tests) in the self-guided group, 16 (81 pretest, 30 post-tests) in the web-assisted group, and 15 (60 pretests, 32 post-tests) in the in-person group.

As we began the postintervention phase of the project in 2022, members of the research team developed a semistructured interview protocol by which we could better elicit participants’ experiences, perspectives, and intentions for adaptation in their cities. The research team began outreach for interview data collection in May 2022, when some participating cities had completed postintervention survey data collection. Interview data collection lasted through August 2023, which enabled data collection in Florida cities where postintervention survey data collection was halted due to the effects of 2022's Hurricane Ian (for more details, see the Supplementary information). Four members of the research team conducted interviews lasting 45 minutes via Zoom with 43 participants from 21 cities in the experiment, as well as three additional cities using FWC outside of the experimental context; four interviews included more than one participant, for 36 total interviews. The interview protocol is included in Supplementary information.

### Ethics

All procedures were approved by the Institutional Review Boards at Stanford University [IRB-2 #54184], University of Oklahoma [IRB #12112], and the University of Michigan [IRB #HUM00170743]. After inviting participants to participate in the study, those interested in participating were provided with details about the research, expectations of participants, and risks and benefits of participating. We then obtained verbal (if participation conducted remotely) or written (if participation in person) consent to participate in the study from the participants.

### Intervention

Participating cities were randomly assigned to one of the three groups that varied by level of engagement: self-guided (least engagement), web-assisted, and in-person (highest cost of engagement).

#### All groups

All participating cities received access to FWC, a web-based tool in which participants are supported to complete a vulnerability assessment of their stormwater system. FWC guides users through the discrete and iterative steps of completing a vulnerability assessment, including incorporating participants’ local expertise with supplemental information developed for each participating city. Features of the tool include multimedia content using text, videos, and maps. Participants engaged with the tool by responding to tool prompts with text input. Participants progressed in the tool by first establishing baseline conditions in their city for weather and climate, social vulnerability, and local capacity. Then, participants were guided to select stormwater system components to assess for their city's system. Participants selected components from a dropdown list of 27 conventional (e.g. outfalls, culverts, treatment facilities, pumps, and pump stations) and 25 low-impact design/nature-based components (e.g. bioswales, floodplains, rivers, and streams). Participants could pick as many or few components as were relevant to their system, and participants in the web-assisted and in-person groups selected at least two components as part of the engagement. For each selected component, participants progressed through tool prompts regarding the component's sensitivity, defined within the tool as “the degree to which an actor, organization, or system can be directly or indirectly affected by weather events, climate conditions, and natural hazards.” Participants then responded to a series of prompts regarding the component's adaptive capacity, defined within the tool as “the ability of actors, organizations, or systems to adjust to and mitigate extreme weather events, climate conditions and natural hazards.” For each section on sensitivity and adaptive capacity, the exercise culminated in participants rating the selected component on a 5-point scale, running from lowest sensitivity to highest and lowest adaptive capacity to highest. Those components which ranked highest on adaptive capacity and lowest on sensitivity were thus assessed as having the lowest vulnerability, and those components which ranked lowest on adaptive capacity and highest on sensitivity were assessed as having the highest vulnerability. Taken together, all evaluated components could be compared for vulnerability across a sensitivity-adaptive capacity vulnerability assessment matrix (Fig. 2). Participants can then share the results of their vulnerability assessment, including the matrix and text responses for each prompt within each component, as a full PDF report. More about FWC can be found here: https://floodwisecommunities.org/.

**Figure 2:**
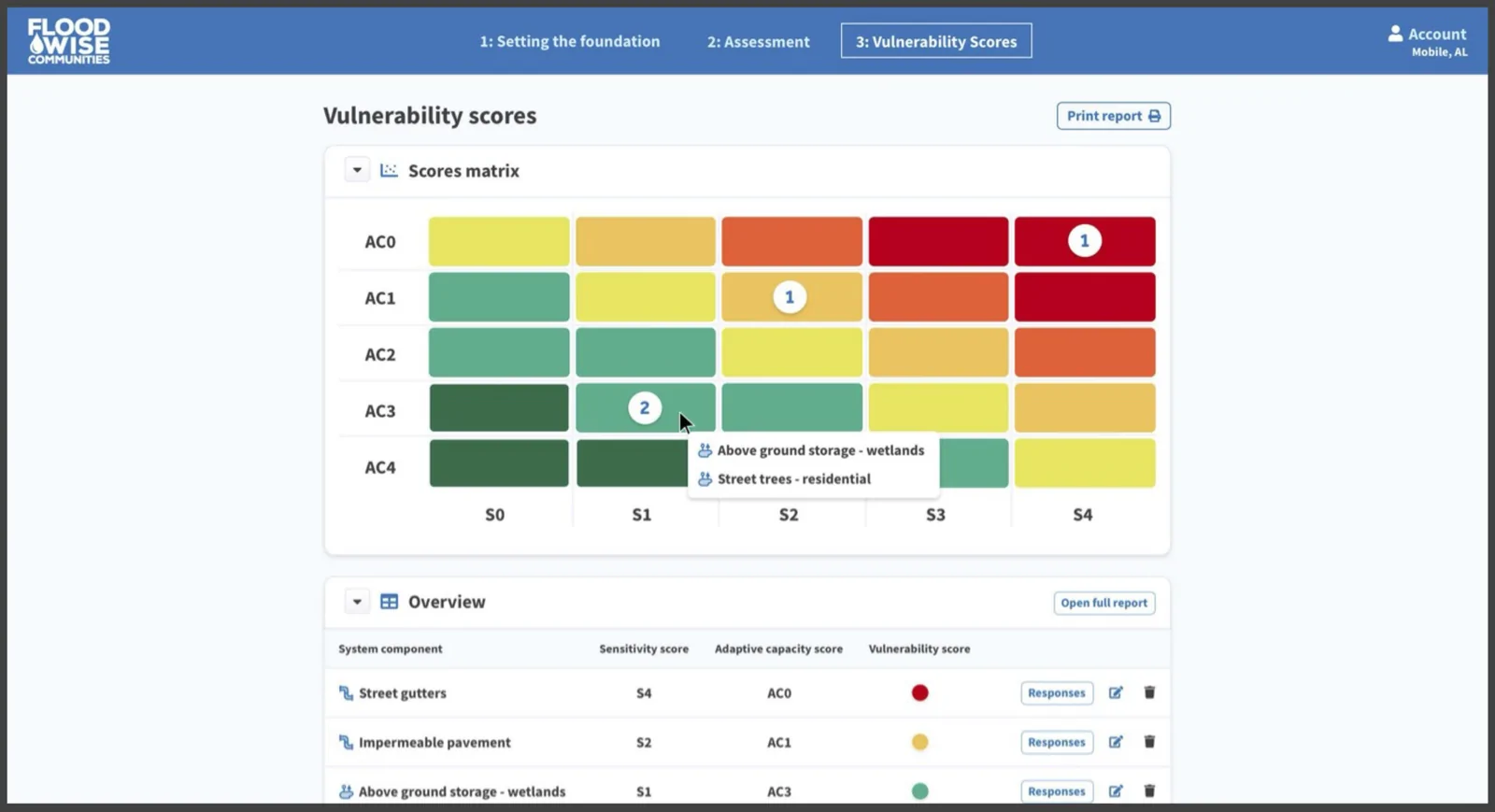
Screenshot of FWC tool vulnerability assessment matrix.

More details about the web-assisted and in-person engagements are included in the Supplementary information.

### Measures

#### Fit

Fit was assessed preintervention and postintervention by averaging agreement with four statements: (i) I [pre: will/post: can] use the tool to support the decisions I need to make, (ii) Data in the tool [pre: will be/post: is] applicable to my community's concerns, (iii) The vulnerability assessment process [pre: will fit/post: fits] with the way I make decisions, (iv) I [pre: will/post: can] apply data from the tool about my community's projected climate impacts to the work I do, (v) I [pre: will be/post: am] familiar with the ways decision-making in my community can incorporate data about my community's social and economic characteristics. All response options were on a 5-point scale, where 1 = strongly disagree and 5 = strongly agree. Cronbach's alpha for fit preintervention (0.91) and postintervention (0.89) suggests good reliability.

#### Legitimacy

Legitimacy was assessed preintervention and postintervention by averaging agreement with two statements: (i) Decisions informed by the tool will be better accepted by our community than those informed by other tools and (ii) Our community will trust projects informed by the information provided by the tool more than other projects. All response options were on a 5-point scale, where 1 = strongly disagree and 5 = strongly agree. Cronbach's alpha for legitimacy preintervention (0.81) and postintervention (0.87) suggests good reliability.

#### Credibility

Credibility was assessed preintervention and postintervention by averaging the rating of each of the following partners: (i) University researchers, (ii) NOAA's Regional Integrated Sciences and Assessments program, (iii) nongovernmental organizations, and (iv) practitioners. All response options were on a 5-point scale, where 1 = not credible at all and 5 = completely credible; “don’t know” responses were coded as 99. Cronbach's alpha for credibility preintervention (0.88) and postintervention (0.90) suggests good reliability.

#### Interplay

Interplay was assessed preintervention and postintervention by averaging the agreement with three statements: (i) The tool [pre: will offer/post: offers] new information from what I already know, (ii) The tool [pre: will provide/post: provides] something different from other tools my department uses, and (iii) My department will use the tool in combination with other tools we already have to support decision making. All response options were on a 5-point scale, where 1 = strongly disagree and 5 = strongly agree. Cronbach's alpha for interplay preintervention (0.77) and postintervention (0.77) suggests good reliability.

#### Usefulness

Usefulness was assessed preintervention and postintervention by averaging ratings of usefulness regarding FWC (pre: “how much do you anticipate”/post: “how much did you”): (i) Identify components of your stormwater system most susceptible to hazards, (ii) identify components of your stormwater system most capable of adapting to hazards, (iii) find relevant climate data about your community, (iv) find relevant socioeconomic data about your community, (v) identify communities that need higher investment to avoid hazards, (vi) communicate importance of climate change to colleagues and officials, (vii) help coordinate different departments in your community, (viii) guide future investment for your stormwater system, (ix) apply for grant funding, (x) develop, change, or update climate action plans, (xi) assist in implementing adaptation interventions, (xii) other [open text field]. All response options were on a 5-point scale, where 1 = not at all and 5 = completely. Cronbach's alpha for use preintervention (0.92) and postintervention (0.93) suggest good reliability.

#### Intended adaptation actions

Intended adaptation actions were broadly defined as being either an enabler to subsequent adaptation action or a direct adaptation action and were assessed preintervention and postintervention by asking participants to indicate which of the following actions their community had or were planning on taking: (i) gathering more information about hazards, (ii) tidal barriers, (iii) natural or green infrastructure projects, (iv) building new levees or sea walls, (v) installing backflow prevention devices (e.g. gates or check valves), (vi) dry flood-proofing (e.g. watertight doors), (vii) Wet flood-proofing (e.g. submersible pump stations), (viii) retreating from shorelines, (ix) relocation of assets, (x) open space preservation, (xi) urban forestry, (xii) low-impact development projects (e.g. grass swales, wet or dry ponds, green roofs, permeable pavements, etc.), (xiii) installing larger pipes, (xiv) maintaining drainage ditches and pipes for higher run off, (xv) raising stormwater inlets, (xvi) installing pumps to force out water, (xvii) building additional conveyance or treatment capacity, (xviii) installing detention or retention ponds to control runoff, (xix) other stormwater retrofits not mentioned above, and (xx) other measures not included above. Selected items were coded as 1 and unselected items were coded as 0. Count scores were created by summing across selected actions.

#### Adaptation-relevant personnel

ARP was ascertained by asking participants to indicate which of the following functions aligned with their role: (i) identify components of your stormwater system most susceptible to hazards, (2) identify components of your stormwater system most capable of adapting to hazards, (iii) find relevant climate data about your community, (iv) find relevant socioeconomic data about your community, (v) identify communities that need higher investment to avoid hazards, (vi) communicate importance of climate change to colleagues and officials, (vii) help coordinate different departments in your community, (viii) guide future investment for your stormwater system, (ix) apply for grant funding, (x) develop, change, or update climate action plans, and (xi) assist in implementing adaptation interventions. Selected items were coded as 1 and unselected items were coded as 0. ARP were defined as those participants who performed three or more of the functions in their role.

#### State

Cities were recruited from the following states: Alabama, Florida, Louisiana, Mississippi, or Texas.

### Data analysis

Descriptive statistics and correlational analyses were conducted. We report means for preintervention and postintervention adaptation actions, fit, legitimacy, credibility, interplay, and usefulness (H1).

In addition, we conducted multilevel models with engagement groups predicting changes over time regarding intended adaptation actions, fit, legitimacy, credibility, and interplay. Tests were two-sided and account for community (participants nested within communities assigned to group) and state (included as a covariate). Random effects for community (unit of randomization) and participant (unit of survey observation) are reported, along with residual errors. Models of legitimacy and credibility for which we observe minimal clustering effects (community-level variance close to 0) are noted in Supplementary information. Models were constructed appropriate to the outcome variable, such that count scores for adaptation are estimated with a Poisson model, and scaled outcomes for fit, legitimacy, credibility, interplay, and usefulness are estimated with linear functional forms. Results include *z*-statistics and effect sizes reported as IRR for Poisson models and standardized coefficients β* for linear models. Degrees of freedom (df) vary by coefficient based on cluster sizes and random effects structure. The reference engagement group for all models is self-guided.

Analyses included ARP as a covariate (H2). We interpret the interaction of ARP and postintervention time for this hypothesis test.

Multiple imputation by chained equations was used to impute missing responses (i.e. skips and refusals) on otherwise complete surveys when appropriate for the intended analysis. Where multiple imputation is used for MLMs (H1, H2), we pool the results of the 20 imputed datasets and report pooled coefficients, IRR, β*, z-statistics, *P*-values, and degrees of freedom.

The research team, including three of the team members who had conducted interviews, began developing the codebook for interview analysis in March 2024 following a deductive approach, in which a priori research questions guided the definitions of features in the codebook. In particular, we focused on the expanded definition of use and nonuse across dimensions of instrumental, conceptual, and symbolic use, as well as the inclusion of levels of actionable knowledge indicators. Upon completing the codebook, two coders who had neither collected interview data nor participated in the original codebook development trained on a subset of interviews in May 2024 (*n* = 5, ∼14% of full corpus). During the training period, coders were encouraged to expand on codebook definitions based on the examples they found in the training set. See Table S14 for the codebook.

Coders labeled parent and child themes using NVivo 14, and training interviews were coded independently by each coder for themes at the paragraph level. Interrater reliability tests were run on the training subset to test for Cohen's kappa (satisfactory initial agreement >0.75). No a priori kappas were satisfied after initial training; kappas ranged from 0.34 to 0.62. Coders met to reach consensus on interviews where they disagreed and revised the codebook to increase clarity going forward, including identifying exclusion criteria.

After training was completed and the codebook updated, coders independently coded the remaining corpus (31 interviews). Analysis proceeded by evaluating descriptive statistics that quantify the prevalence of themes in interviews and their colocations.

## Supplementary Material

pgag330_Supplementary_Data

## Data Availability

The survey data supporting the findings reported in this manuscript can be found at: https://doi.org/10.7266/nsa3qzmk. Deidentified interview data supporting the findings reported in this manuscript (freely available at: https://data.griidc.org/data/T5.x896.000:0001). Survey data analysis was conducted using scripts coded for R v4.4.0. Code is available in the OSF project at https://osf.io/r2qwe/.
