# Supplementary material for: Building adaptive capacity of US Gulf Coast city practitioners to extreme weather events
Source: PNAS Nexus. 2026 Sep 28;5(10):pgag330. doi: 10.1093/pnasnexus/pgag330 (PMC13650718; doi:10.1093/pnasnexus/pgag330)
Supplement: Supplementary file 1 [file pgag330_supplementary_data.pdf]

Supplementary Information
Title
Building adaptive capacity of US Gulf Coast city practitioners to extreme weather events

SI Figure S1. Geographic distribution of participating cities.

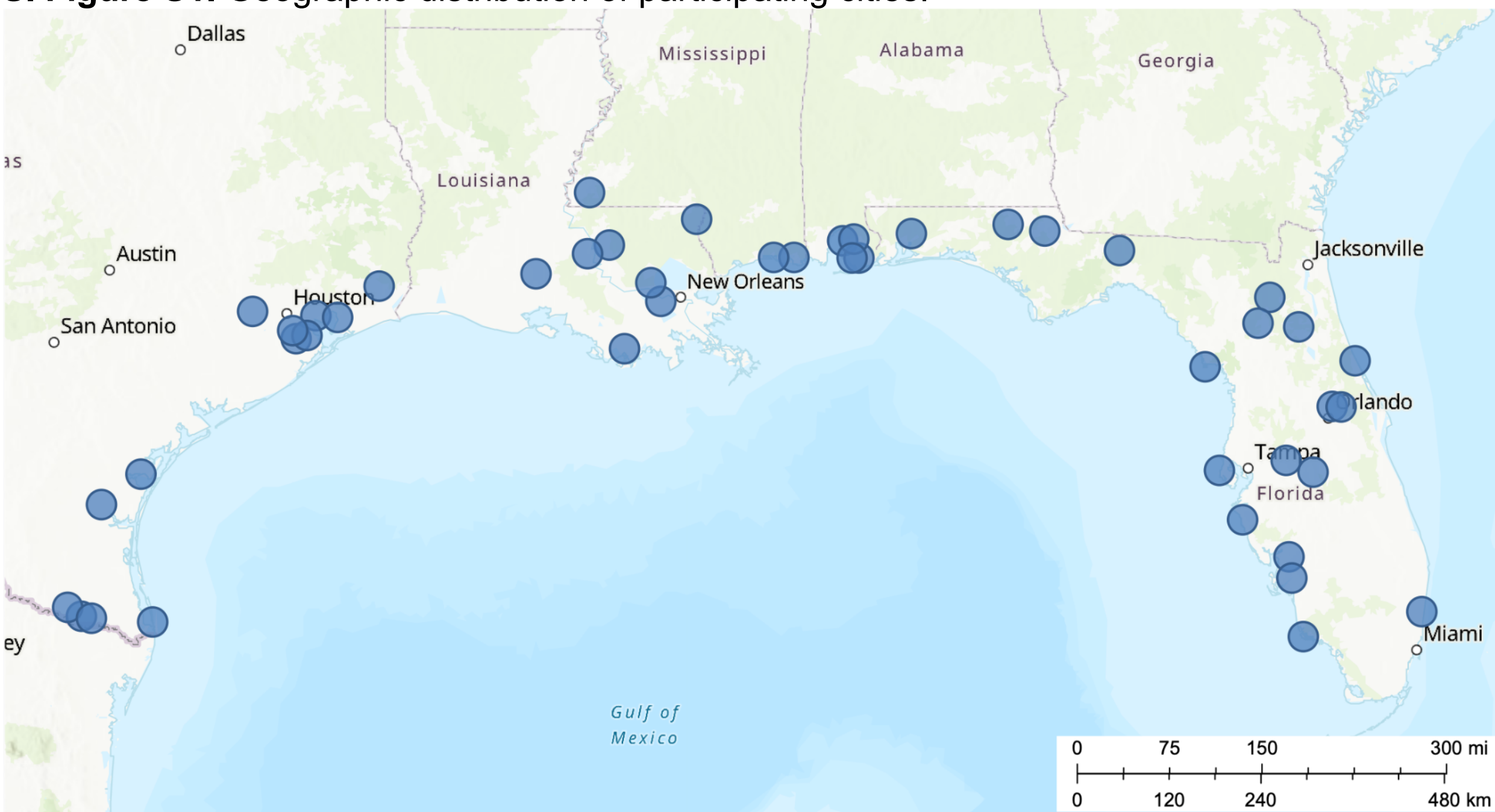

Legend: Points indicate location of cities included in the study sample across Texas, Louisiana, Mississippi, Alabama, and Florida.

SI Table S1. Characteristics of participating cities by treatment group

Characteristic		Overall (n=46)	In-person (n=15)	Self-guided (n=15)	Webinar (n=16)	p-value
Number of participants, median [IQR]		4.00 [3.00, 5.00]	3.00 [3.00, 5.50]	4.00 [3.50, 4.50]	5.00 [4.00, 5.25]	0.151
Population, median [IQR]		34812 [16820, 81106]	18198 [3235, 69187]	47319 [24547, 97874]	41319 [24687, 59462]	0.122
Social Vulnerability Index, median [IQR]		0.72 [0.47, 0.91]	0.74 [0.68, 0.86]	0.66 [0.41, 0.90]	0.67 [0.45, 0.96]	0.804
State, n (%)						0.957
	AL	4 (8.7)	2 (13.3)	1 (6.7)	1 (6.2)	
	FL	19 (41.3)	5 (33.3)	7 (46.7)	7 (43.8)	
	LA	7 (15.2)	2 (13.3)	2 (13.3)	3 (18.8)	
	MS	3 (6.5)	2 (13.3)	1 (6.7)	0 (0.0)	
	TX	13 (28.3)	4 (26.7)	4 (26.7)	5 (31.2)	

Note: Columns show characteristics for the full sample and by treatment group. Values are medians [interquartile ranges] for continuous variables and n (%) for categorical variables. Kruskal–Wallis tests were used for continuous variables and Fisher’s exact tests for categorical variables. P-values are two-sided. The observed characteristics were similar across treatment groups.

City-level adaptive capacity

Research suggests adaptive capacity must be available in both generic (e.g., general health, wealth, education, political stability) and specific (e.g., disaster warning systems, climate-proofed infrastructure, decentralized power grids) forms. Taken together, generic and specific forms of adaptive capacity can shape a city's adaptability to challenges posed by structural deficits (e.g., poverty and injustice) and climate change-driven events (e.g., severe storms and drought)[55]. Building adaptive capacity at the city scale requires the involvement of different actors, institutions and organizations, knowledges, and scales of decision-making and governance. In this conceptualization, broader institutional structures at the city level – which in turn depend on other levels of decision-making and resources such as the state and federal governments – build general capacity through the mobilization of "capitals" (e.g., financial, educational, social, cultural, ecological, institutional, political and informational, among others[20]) and uphold adaptation rules and norms. In addition, specific sectors within cities (e.g., emergency response, stormwater management, hazards management, sustainability planning, among others) are tasked with building capacity to specific stressors (e.g., flooding, heat and cold events, drought, sea-level rise) The combination of these capacities, in turn, create an iterative dynamic process where all actors, sectors, and hierarchical levels are reflexively responding to each other and building adaptive capacity[55–57] across socio-ecological and infrastructural systems to prepare, respond, and adapt to climate-driven stressors[55]. Figure S2 shows perceived city-level capacity to manage and withstand impacts.

SI Figure S2. Perceived city-level capacity to manage and withstand impacts (n=200, 46 cities).

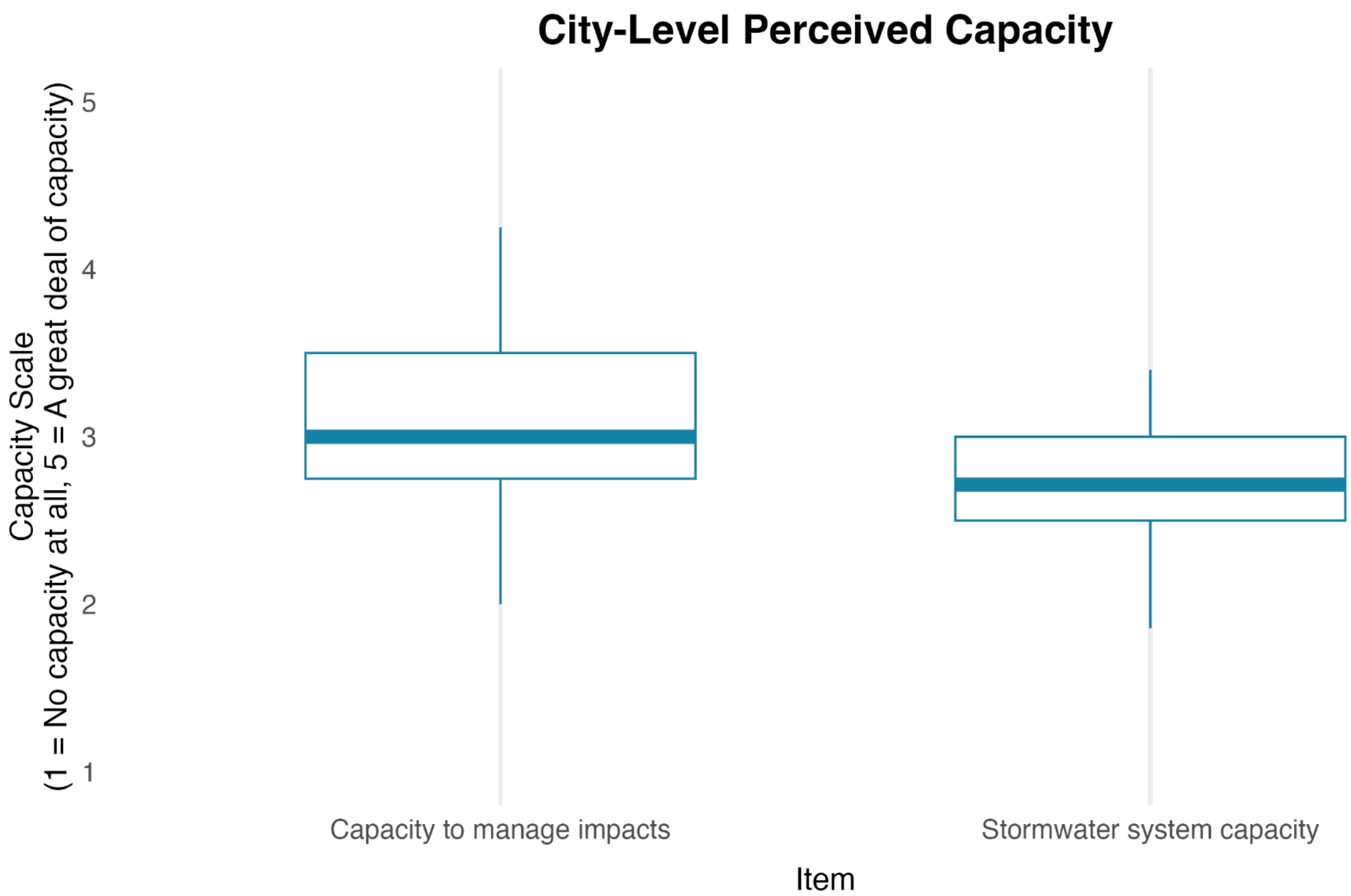

Legend. Tukey-style boxplots in which the vertical lines within each box represent the median value and box limits are interquartile range (IQR) for each item. Lower whiskers include values greater than or equal to the first quartile minus 1.5×IQR; upper whiskers include values less than or equal to the third quartile plus 1.5×IQR. Source: pre-test surveys (n=200).

Description of FWC

A cornerstone of FWC is the historical and projected climate data compiled for participants by our team of climatologists for this study[34]. Participating cities received the four-chapter report "Climate of the Gulf Coast States,"[35] which includes: a historical summary of the Gulf Coast's climate; a review of historical changes connected to scientific consensus on anthropogenic climate change; an explanation of climate modeling, including discussion of how climate model data are used in the intervention and what changes to the Gulf Coast are expected by end-of-century; and summaries of hazards expected with climate change (e.g., sea level rise, hurricanes, floods) and the underlying climatology behind expected changes. In addition to this comprehensive report, each city received a "Weather and Climate in [city]" report, a four-page digest of downscaled climate projections for the participating city that focuses on historical and projected changes in temperature (e.g., extreme heat) and precipitation (e.g., heavy rainfall days), as well as connecting these changes to recent severe weather events in each location. An example "Weather and Climate" report is included below (SI Figure S3).

FWC also integrates into the assessment another web-based tool, Neighborhoods at Risk. Developed by Headwaters Economics, Neighborhoods at Risk visualizes Census data for cities to incorporate social vulnerability characteristics at the census tract level. Each participating city

received training on how to use the socioeconomic data mapping tool and interpret data profiles generated from this tool. Training used the tool's automated assessment of increased social vulnerability and climate risk, and participants were trained on how to explore additional variables and localized data and generate reports on their own. More information about the Neighborhoods at Risk tool can be found here:
https://headwaterseconomics.org/tools/neighborhoods-at-risk/tool-about/

SI Figure S3. A sample “Weather and Climate Report” created for Orleans Parish, LA (generated as an example, not a participating FloodWise Community).

FLOOD WISE COMMUNITIES

Weather and Climate in Orleans Parish, Louisiana

Overview

Orleans Parish is highly vulnerable to being flooded, whether due to high tides, heavy rain, or the storm surges and rainfall of passing hurricanes, most famously Hurricane Katrina in 2005. Much of Orleans Parish sits below sea level, hence its heavy reliance on the tall levees surrounding it; a breach of these levees can cause severe flooding across the entire parish. Changes to Orleans Parish’s future rainfall totals are uncertain, but rapid sea level rise (2.5 feet in the last 70 years) is raising the risk of the parish’s levees failing more frequently, thus also raising the risk of future flooding.

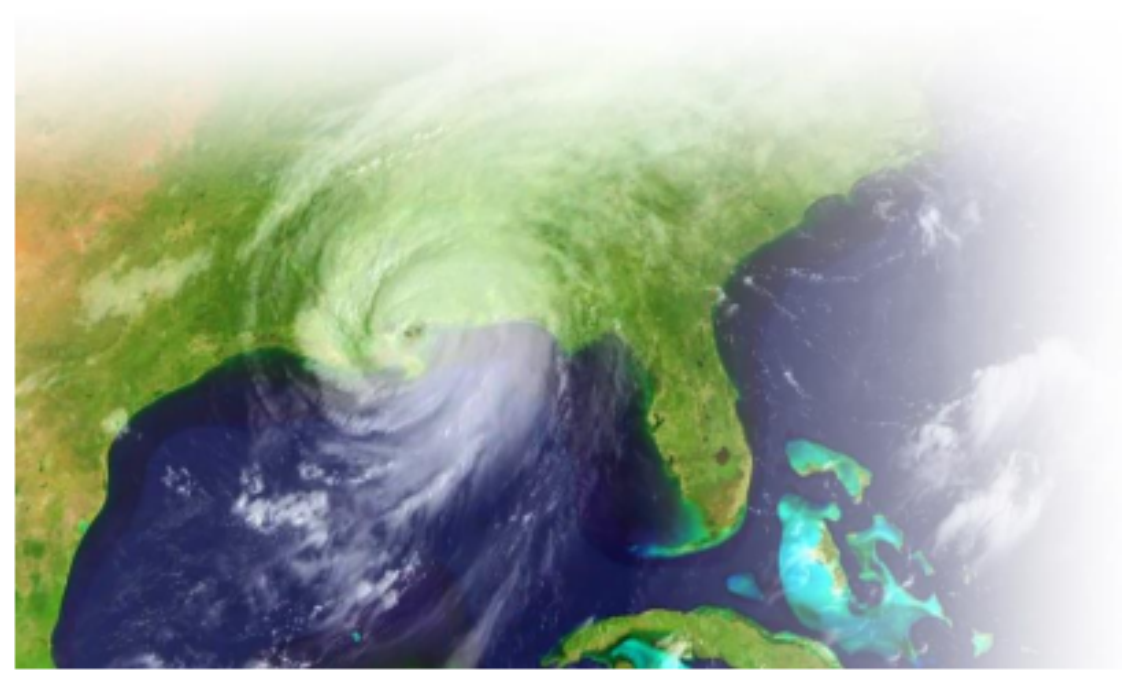

Hurricane Katrina (CC BY 2.0).

Temperatures in Orleans Parish have increased notably since the 1990s, whereas its rainfall totals have declined. Climate models suggest a move toward slightly wetter future conditions, though with high uncertainty. Key statistics about recent and future climate change in Orleans Parish are given in the *Quick Climate Facts* table (right); see the following pages for further details.

Climate Change Along the Gulf Coast

The Gulf Coast has seen increased temperatures and decreased average rainfall, but occurring in heavier downpours, in recent decades. The region’s coastal wetlands and low-lying cities are especially vulnerable to the challenges presented by climate change, such as reduced freshwater supply, more active hurricane seasons, and increased tidal flooding from sea level rise.

As global temperature increases continue, adapting to these challenges will be essential for the sustainability of communities along the Gulf Coast. Further details can be found in the *Climate of the Gulf Coast States* report, prepared by FloodWise Communities.

Quick Climate Facts

	Current (1991-2020)	Future (2071-2100)
TEMPERATURE	***It’s getting hotter…***<br>**+2.4°F** *increase of average annual temperature*<br>**80 nights** *above 75°F each year (average)*<br>**33 days** *above 95°F each year (average)*	***Future temperatures could be even higher…***<br>**Winter:** *4 to 8°F warmer*<br>**Spring:** *4 to 8°F warmer*<br>**Summer:** *6 to 9°F warmer*<br>**Autumn:** *6 to 12°F warmer*<br>**>64 extra days** *above 95°F each year*
RAINFALL	***It’s getting drier…***<br>**-6.3 in** *decrease of average annual rainfall*<br>**5 days** *of heavy rainfall (>2 in) each year (average)*	***Future rainfall is less certain…***<br>**Winter:** *-3 to +5 in*<br>**Spring:** *-4 to +3 in*<br>**Summer:** *-5 to +6 in*<br>**Autumn:** *-1 to +4 in*<br>**-1 to +3 extra days** *of heavy rainfall each year*

Data sources: SC-ACIS; NA-CORDEX.

SI Figure S3 Continued.

Weather and Climate Profile - Orleans Parish, Louisiana

Key Details

- **Average temperatures in Orleans Parish increased by 2.4°F between 1991 and 2020.**
- **Higher emissions scenarios could raise temperatures between 5.8 and 9.1°F by 2100.**
- **Orleans Parish saw its average rainfall decline by -6.3 in over the last 30 years.**
- **Future rainfall over Orleans Parish is uncertain, but leans toward wetter conditions.**
- **Orleans Parish's levees are increasingly likely to fail as sea levels continue to rise.**

Temperature in Orleans Parish

Orleans Parish's climate is humid-subtropical, meaning its summers are warm and its winters are mild. Annual average temperatures in the parish increased by 2.4°F since the 1990s, which has particularly affected nighttime conditions, hence the increase in its annual average low (+3.2°F) and the number of warm nights (>75°F) per year (+55 days). Temperatures across Southeast Louisiana have been increasing since the late-1970s, at an especially fast rate during the 2010s (right). If this rate of increase continues, temperatures in Orleans Parish could increase by another 9.1°F by 2100.

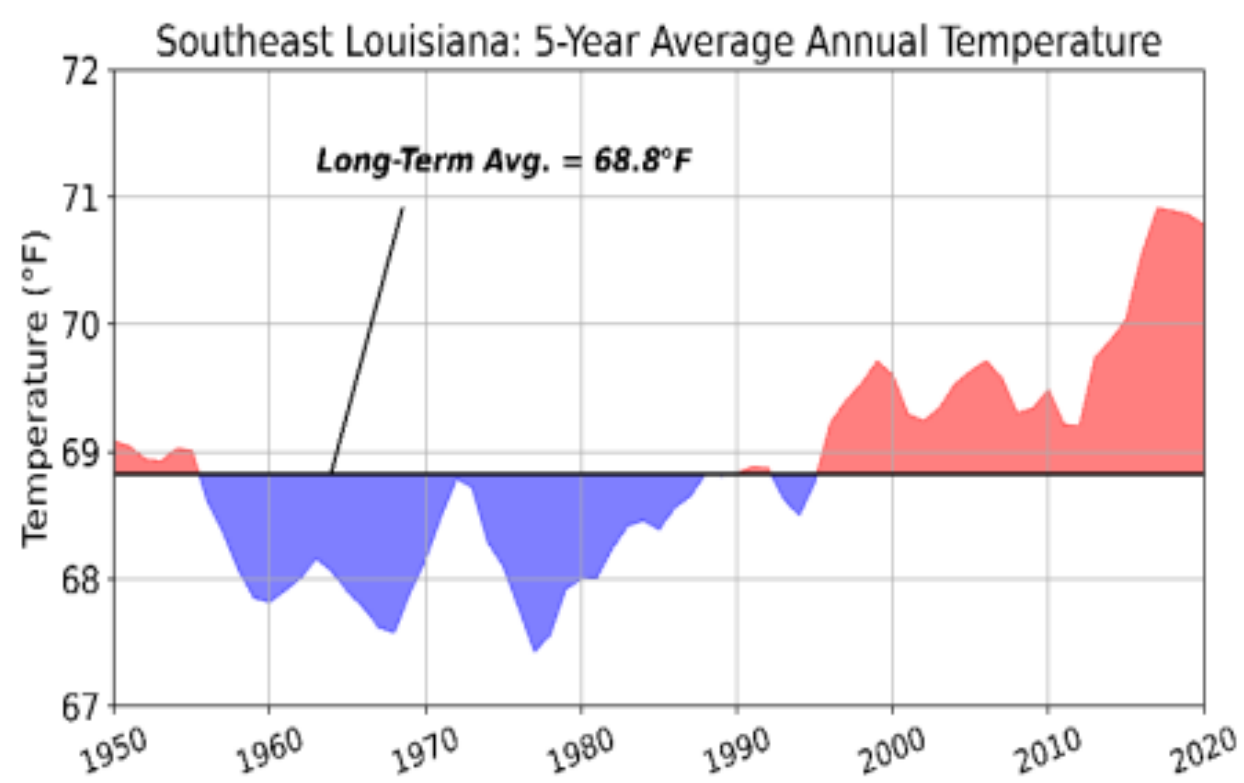

Data Source: SRCC.

Temperatures in Southeast Louisiana have been increasing since the late-1970s.

Historical and Projected Temperature – Orleans Parish						
Variable	Historical Average: 1991-2020	Historical Change: 1991-2020	Mid-Century Projections: 2041-2070*		End-Century Projections: 2071-2100*	
			Min	Max	Min	Max
Annual Avg Temp.	71.1°F	+2.4°F	73.6°F	76.6°F	76.9°F	80.2°F
Winter Avg Temp.	56.8°F	+1.3°F	59.2°F	61.2°F	61.2°F	64.6°F
Spring Avg Temp.	70.9°F	+2.9°F	73.6°F	76.0°F	75.2°F	78.4°F
Summer Avg Temp.	84.2°F	+1.8°F	86.9°F	90.3°F	90.0°F	92.8°F
Autumn Avg Temp.	72.1°F	+3.0°F	74.6°F	78.2°F	78.2°F	84.4°F
Annual Avg High	79.8°F	+1.7°F	82.3°F	85.3°F	85.5°F	89.3°F
Annual Avg Low	62.4°F	+3.2°F	65.3°F	67.7°F	68.2°F	72.0°F
Hot Days (>95°F) per year	33 days	+14 days	61 days	95 days	97 days	147 days
Warm Nights (>75°F) per year	80 days	+55 days	143 days	169 days	175 days	211 days

Data sources: SC-ACIS; NA-CORDEX.
** Projections represent the range of highest and lowest projections from the NA-CORDEX climate models, driven by the RCP 8.5 emissions scenario.*

SI Figure S3 Continued.

Weather and Climate Profile - Orleans Parish, Louisiana

Rainfall in Orleans Parish

Orleans Parish is wet year-round, hence its high annual average total (60.2 in), though summer is its wettest season (21.6 in). The annual total has declined over the last 30 years (-6.3 in), and has also fallen less often in heavy bursts (-1 heavy rain day per year). A shift toward drier conditions since the 1990s has similarly happened across Southeast Louisiana (right), though still part of its regular wet and dry period cycle. Future changes to rainfall totals in Orleans Parish are uncertain, but lean toward wetter conditions.

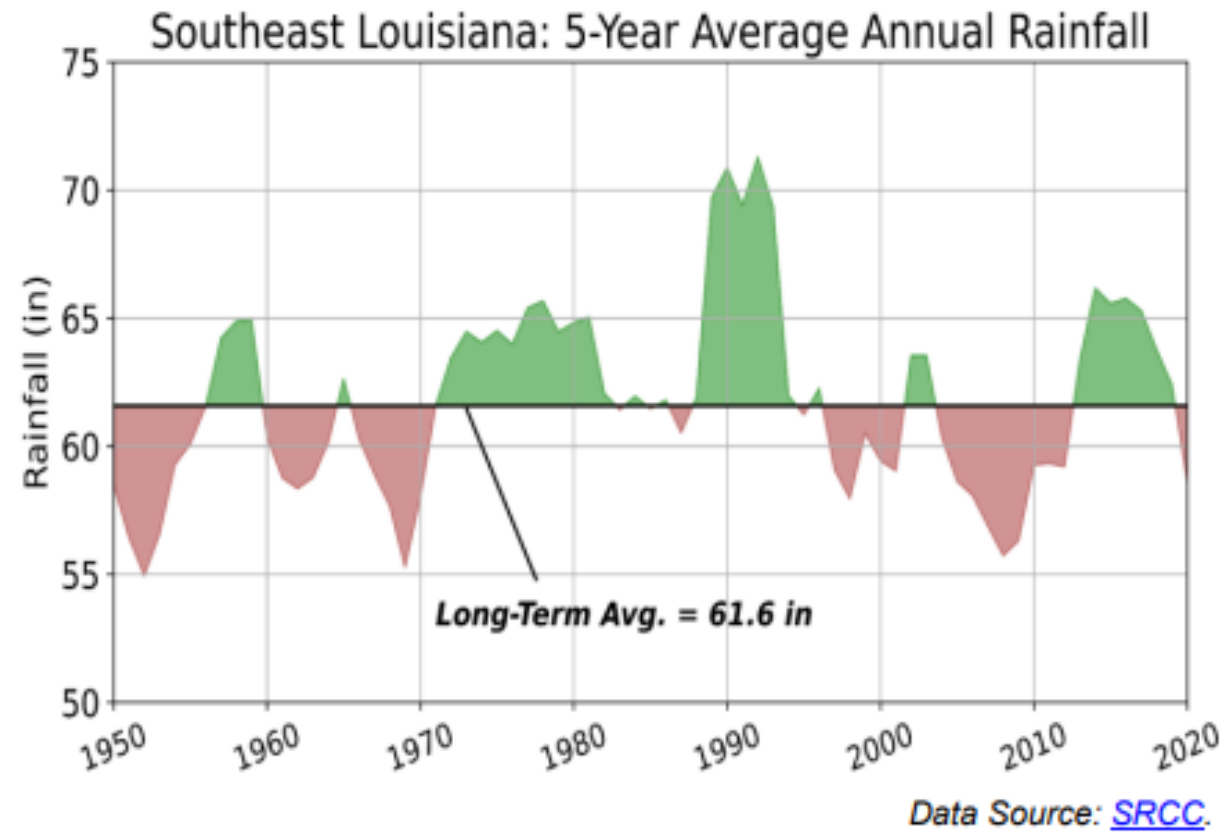

Data Source: SRCC.

Recent years in Orleans Parish have been drier than average, though how rainfall totals will change in future decades in uncertain.

Historical and Projected Rainfall – Orleans Parish						
Variable	Historical Average: 1991-2020	Historical Change: 1991-2020	Mid-CenturyProjections: 2041-2070*		End CenturyProjections: 2071-2100*	
			Min	Max	Min	Max
Annual Avg Rainfall	60.2 in	-6.3 in	53.4 in	74.8 in	49.9 in	70.5 in
Winter Avg Rainfall	13.0 in	-5.8 in	11.3 in	16.7 in	10.2 in	17.8 in
Spring Avg Rainfall	13.6 in	-2.3 in	11.1 in	17.9 in	9.8 in	16.6 in
Summer Avg Rainfall	21.6 in	+3.2 in	17.9 in	29.7 in	16.5 in	27.3 in
Autumn Avg Rainfall	13.2 in	-2.1 in	10.9 in	17.7 in	11.9 in	16.9 in
Avg Heavy Rain Days (>2-in) per year	5 days	-1 day	5 days	9 days	4 days	8 days

Data sources: SC-ACIS; NA-CORDEX.
** Projections represent the range of highest and lowest projections from the NA-CORDEX climate models, driven by the RCP 8.5 emissions scenario.*

Recent Severe Weather Events in Orleans Parish		
Event	Date	Notes
Orleans Parish Flash Flood	Aug 2017	9 inches of rain fell in 5 hours. Drainage systems blocked up and caused widespread street flooding. Floodwaters damaged hundreds of homes. $5 million in property damages.
Hurricane Isaac	Aug 2012	Roads and homes flooded by a 7 to 9 feet storm surge. Up to 20 inches of rain fell over parts of New Orleans and strong winds lasted for 2 days. $27 million in property damages.
Hurricane Gustav	Sep 2008	Damages from the storm surge were worst on the parish's east side, outside of its levee system. 600 homes affected, mostly by wind damage. $7 million in property damages.
Hurricane Katrina	Aug 2005	A 12 to 15 feet storm surge hit Orleans Parish, flooding 80% of the city itself. Catastrophic damage due to breached levees. 638 deaths and $21 billion in property damages.
Hurricane Cindy	Jul 2005	Heavy damage to trees and power lines by sustained winds over 55mph. Minor damage caused by tides 3 to 5 feet higher than normal. $17 million in property damages.

Data sources: Flood Factor; NOAA NCEI Storm Events Database.

FLOOD WISE COMMUNITIES

https://floodwisecommunities.org Weather and Climate Profile | 3

SI Figure S3 Continued.

Weather and Climate Profile - Orleans Parish, Louisiana

Flood Risk:

Around 150,477 (~100%) properties in Orleans Parish are at risk of flooding.

Almost all properties in Orleans Parish are at moderate flood risk (light-orange). This risk would be higher were it not for the levees that protect the parish from high water levels along the Mississippi River and in Lake Pontchartrain. Flood risk is major (orange) nearer the edges of the river, and extreme (dark-red) on the southeast side of the lake. In 30 years' time, environmental factors such as climate change will increase the number of properties at risk of flooding to around 150,515.

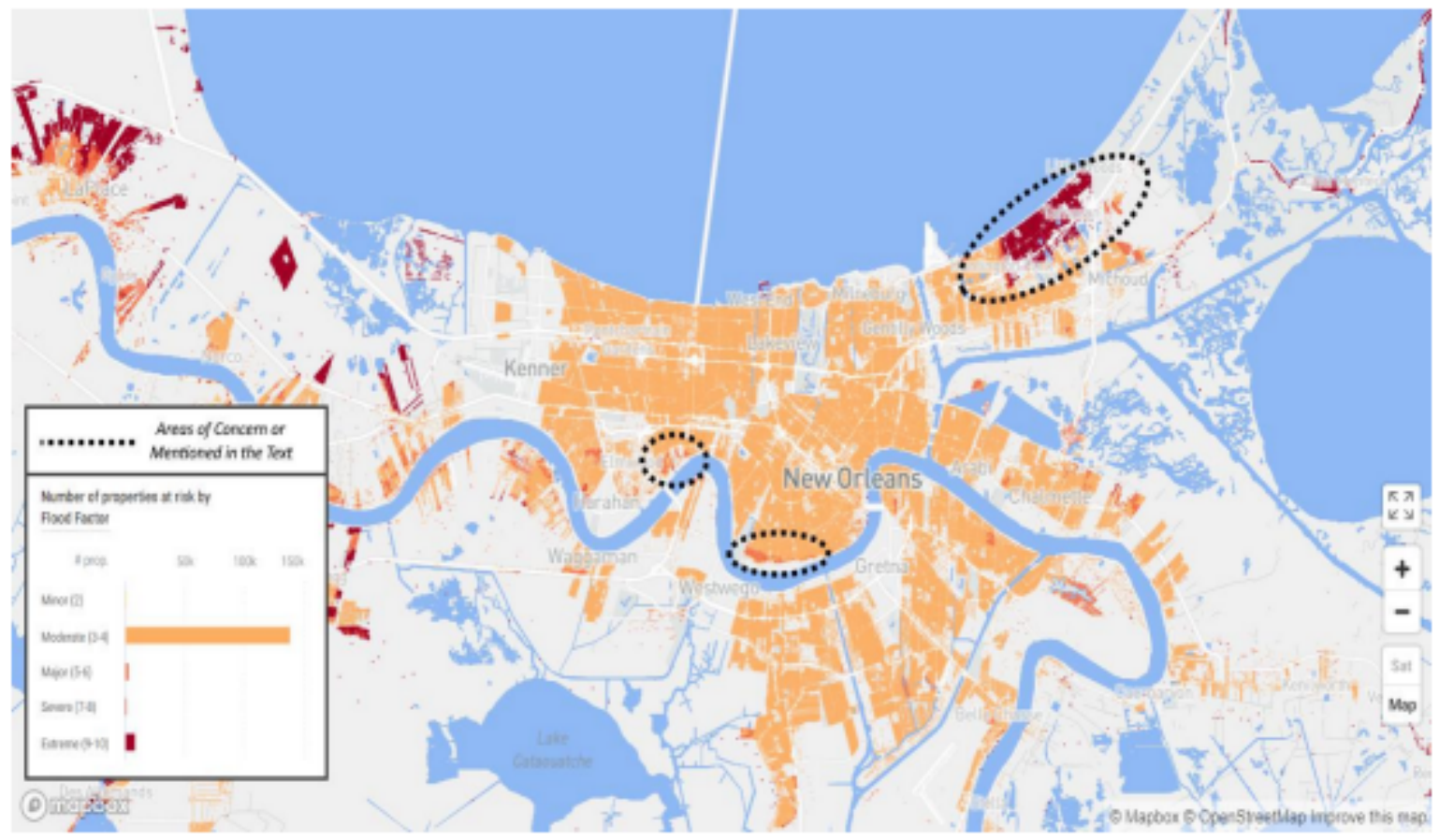

Image Source and more flood risk resources: [FloodFactor]

Sea Level Rise:

A 10-feet rise would almost entirely cut Orleans Parish off from the mainland.

Southeast Louisiana has seen a mean sea level increase of over 2.5 feet in the last 70 years, due to climate change and land subsidence. The worst-case extent for sea level rise (+10 feet by 2100) would drown almost all areas outside of Orleans Parish's levees (even an extra 2 feet would achieve this). The higher water levels would make a breach or damage to these levees much more likely. Additionally, New Orleans Lakefront Airport would be submerged, and only a sliver of land around the Mississippi River would keep Orleans Parish linked to the mainland.

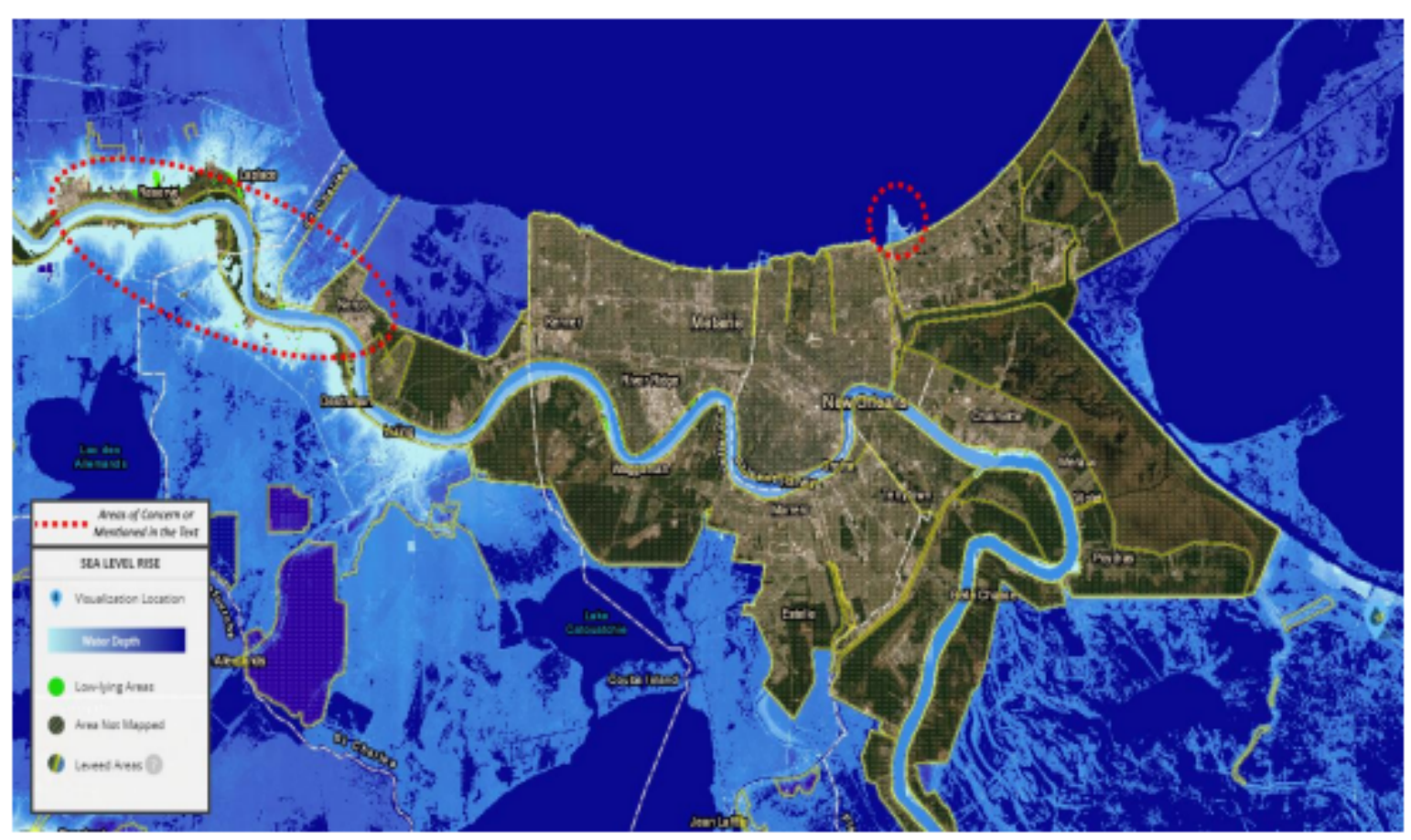

Image Source: [NOAA SLR Viewer]

End of profile. Please visit https://floodwisecommunities.org for more information.

Description of engagement intervention

Self-guided group: Participating cities assigned to receive self-guided access to FloodWise Communities received access after completing pre-test surveys. Tool access included credentials (one credential shared by all members of a FWC team) to use the web-based FloodWise Communities tool, which included written and video tutorials guiding participants step-by-step through the process of identifying, explaining, and ranking the susceptibility and adaptive capacity of local stormwater system components to achieve an overall vulnerability assessment matrix. Within the FloodWise Communities tool was a section on measuring socioeconomic vulnerability, which was supported by the web interactive tool "Neighborhoods at Risk" developed by Headwaters Economics, a partner on the research team. Participants also received two documents regarding climate data developed for them by team investigators from SCIPP at the University of Oklahoma and GLISA at the University of Michigan - two of NOAA's regional CAP/RISA teams. The first document was the multi-chapter report "Climate of the Gulf Coast States: An Examination of Climate Change's Effects across the Region," which uses observations and climate model data to describe historical and projected climate data for the U.S. Gulf of Mexico region. The second document was a "Weather and Climate Profile" generated individually for each community to summarize historic and projected changes in heat and rainfall (see Figure S3 below for example). Both products were developed using SC-ACIS and NA-CORDEX data. Participants in the self-guided condition were informed to contact study personnel with any questions they may have, and participants were asked to email study personnel when they had completed their vulnerability assessment using the FloodWise Communities tool. At this time, study personnel re-contacted participants with post-intervention surveys.

Web-assisted group: Participating cities randomly assigned to the web-assisted group received all facets of the access described above, with the addition of an eight-hour vulnerability assessment webinar engagement led by the research team. Engagements were scheduled with cities at their convenience beginning in October 2021. Trained engagement specialists from the FWC team led workshops using each city's preferred video call platform (e.g., Zoom or Microsoft Teams) and encouraged all participants to have video on. Some cities had team members working together in the same room for the video call, whereas others had team members joining from separate locations via video, in part determined by the city's policy around remote work at the time due to the COVID-19 pandemic. Engagement specialists from the FWC team included two co-leads for the workshop: one an adaptation specialist, the other a scientist. The FWC team also had at least one graduate student join the workshops to provide technical assistance and collect rigorous participant observation notes. Workshop co-leads followed an agenda and used a slide deck template that was shared across all webinar and in-person engagements. Workshops were structured around brief (2-10 minute) lectures by co-leads regarding materials in FloodWise, including weather and climate, social vulnerability, resilience, and adaptive capacity. These brief lectures set the foundation for the majority of participant time using the tool as a team in the workshop. Tool prompts led to team discussions,

with participants variably relying on personal experience, role expertise, and supplementary data from FloodWise to answer prompts. City teams were encouraged to pick three to five components to assess with a mix of conventional and low-impact design components. Once city teams had completed their components assessments and discussed the vulnerability matrix results, co-leads worked with participants to determine next steps that would be most relevant for their city (e.g., assessing vulnerability for more stormwater system components, sharing the vulnerability assessment report with other municipal stakeholders, using vulnerability assessment results to seek grants). Workshops that were split into two four-hour sessions across successive days ended with sensitivity assessments of the first selected component on day one and began with adaptive capacity of the first selected component on day two.

In-person group: Participating cities randomly assigned to the in-person group received all facets of the engagement described above, with the distinction that the eight-hour engagement occurred in person. Engagements were scheduled with cities at their convenience beginning in January 2022. FWC engagement teams traveled to participating cities to complete one- or two-day workshops in facilities of cities choosing, often meeting rooms in municipal offices. Workshops were structured following the same agenda described above. Additionally, the FWC team provided catered breakfast and lunch for participants, and this "non-workshop" time was often an opportunity to connect with each other, especially as guests to the cities.

Observed differences by engagement group

Pre-registered analyses (found at this link: https://doi.org/10.17605/OSF.IO/RJAHY) included hypotheses testing for differences between engagement groups post-intervention as well as a hypothesis testing for the mediating effects of actionable knowledge on reported usefulness and intended adaptation actions. We report the results of these tests by engagement groups here.

Across all levels of engagement, we saw a decline in some indicators of actionable knowledge after FWC (Table S2). When testing for statistical differences, we found significant decreases in perceptions of FWC fit (estimate [β] = -0.705, 95% confidence interval [CI; -1.213, -0.197], standardized coefficient [β*] = -0.502, $z = -2.731$, $p = 0.007$, Table S4), interplay (β = -0.531, 95% CI [-0.943, -0.120], β* = -0.367, $z = -2.543$, $p = 0.012$, Table S7), and usefulness (β = -0.587, 95% CI [-1.104, -0.070], β* = -0.174, $z = -2.234$, $p = 0.026$, Table S8), although the decline was not meaningful (SI Figure S4), as perceptions remained moderately positive pre- versus post-intervention. For the other indicators of actionable knowledge – legitimacy and credibility – we saw no difference in perceptions after intervention.

Those in the web-assisted group expressed higher perceptions of tool fit than those in the self-guided group post-intervention (Fig. S3; β = 0.378, 95% CI [-0.040, 0.796], β* = 0.521, $z = 1.782$, $p = 0.076$, Table S4). A similar pattern was observed for legitimacy, although not statistically meaningful given low community-level variance for this outcome and credibility (Tables ED5-ED6, see notes). No other differences emerged between either in-person or web-assisted on perceptions of actionable knowledge.

Additional registered hypotheses tested whether indicators of actionable knowledge mediated engagement group effects on reported usefulness or intended adaptation actions. We tested this hypothesis with structural equation models (SEM). Direct effects were modeled for the effect of engagement group on post-intervention intended adaptation actions, and indirect effects were modeled for the product of engagement group on hypothesized mediators pre-intervention and hypothesized mediators on intended adaptation actions. All tests were two-sided. Each hypothesized mediator was run in separate models.

We found no evidence that perceptions of fit (SI Table S10), legitimacy (SI Table S11), credibility (SI Table S12), and interplay (SI Table S13) mediated the relationships between engagement level and reported usefulness or intended adaptation actions. This may be due to being underpowered because of attrition or because those who are most engaged with FWC are more likely to continue post-intervention.

SI Figure S4. Pre- versus post-intervention outcomes for (A) intended adaptation actions, (B) tool fit, (C) interplay, (D) usefulness, (E) legitimacy, and (F) credibility.

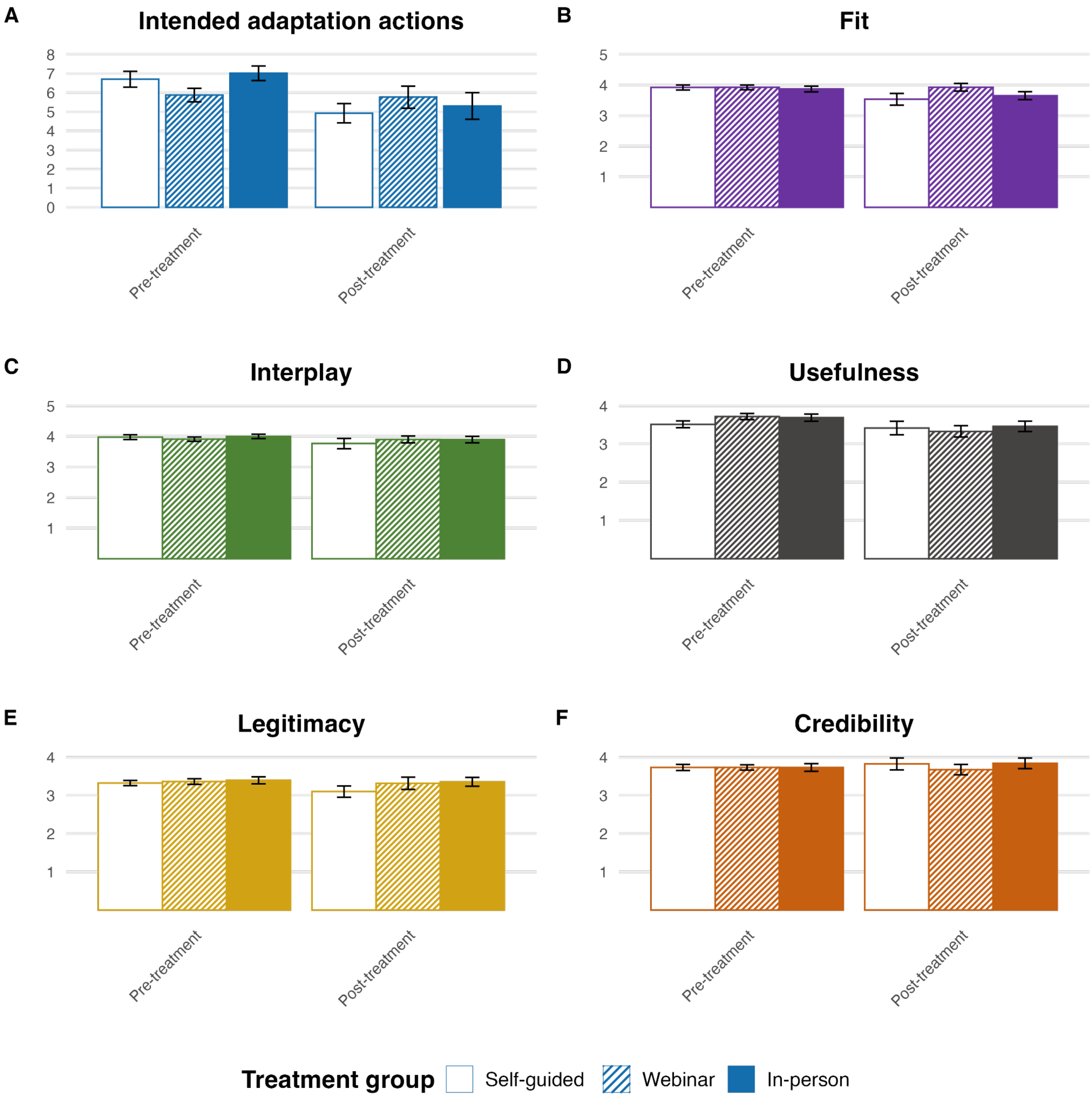

Legend. Error bars indicate standard errors. Across all groups, **(A)** Intended adaptation actions pre-intervention (n=200) mean (M)=6.54, standard deviation (SD)=3.27; post-intervention (n=84) M=5.26, SD=3.26; **(B)** Fit pre-intervention (n=200) M=3.90, SD=0.72; post-intervention (n=81) M=3.73, SD=0.73; **(C)** Interplay pre-intervention (n=198) M=3.97, SD=0.62; post-intervention (n=80) M=3.88, SD=0.62; **(D)** Usefulness pre-intervention (n=194) M=3.65, SD=0.73; post-intervention (n=80) M=3.42, SD=0.81; **(E)** Legitimacy pre-intervention (n=199) M=3.35, SD = 0.65; post-intervention (n=81) M=3.29, SD=0.75; **(F)** Credibility pre-intervention (n=144) M=3.72, SD=0.69; post-intervention (n=62) M=3.75, SD=0.77.

SI Figure S5. (A) City-level availability of capitals for stormwater adaptation (n=200, 46 cities) and (B) city team coverage of roles relevant to adaptation decision-making.

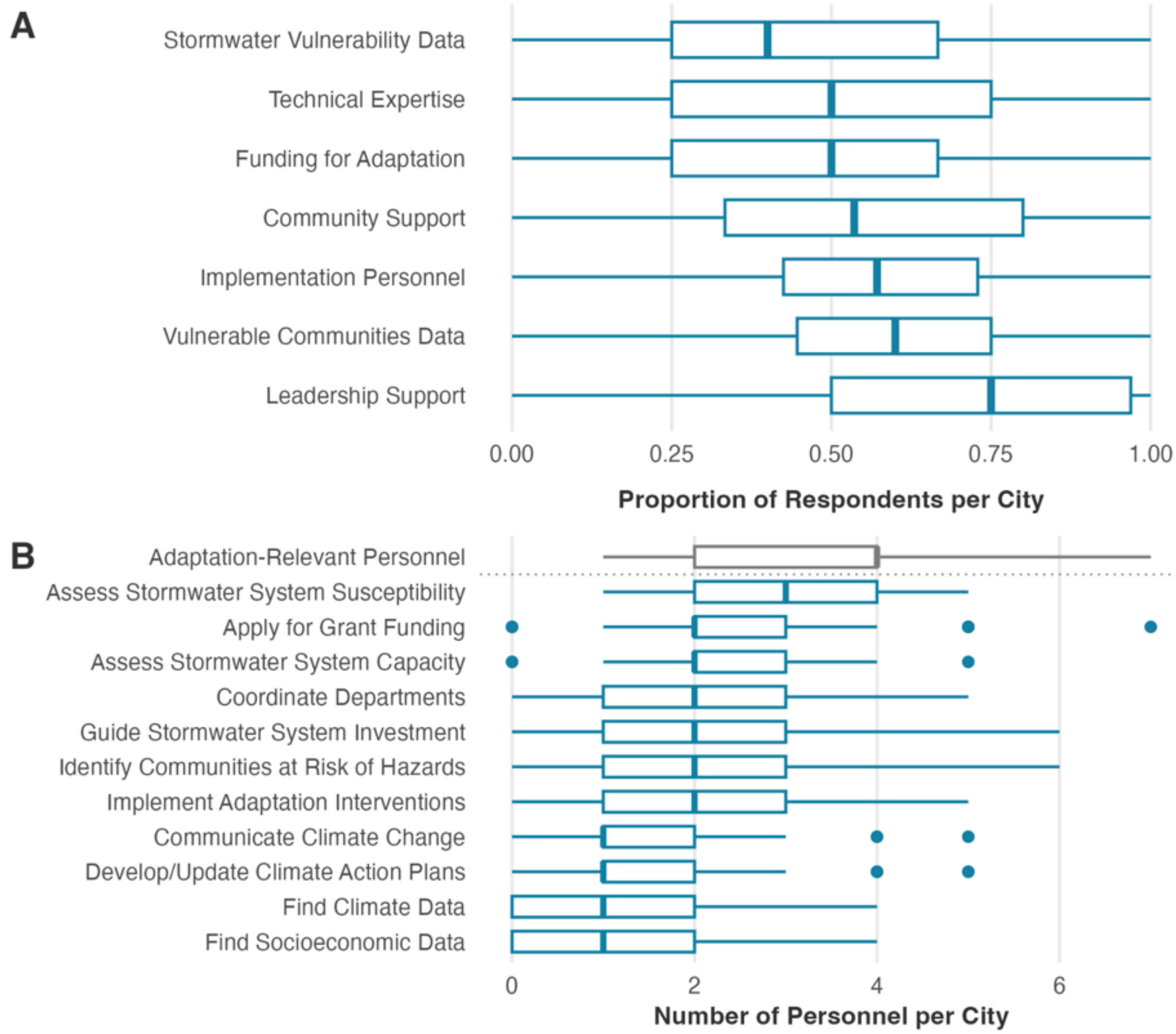

Legend. Tukey-style boxplots in which the vertical lines within each box represent the median value and box limits are interquartile range (IQR) for each item. Lower whiskers include values greater than or equal to the first quartile minus 1.5×IQR; upper whiskers include values less than or equal to the third quartile plus 1.5×IQR. Points beyond whiskers represent outliers, which fall outside the defined 1.5×IQR in either direction from the box limits. Source: pre-intervention surveys (N=200). (A) Units of analysis are city teams. For each adaptation capital, values represent the proportion of participants within a city team who reported that capital was available in their community. For example, a median value of 50% for technical expertise and funding indicates that, in the median city, half of the city team reported access to those forms of capital. (B) Units of analysis are city teams. Values represent the total number of personnel on a city team reporting this role (binary) and are not averaged by the number of practitioners on a city team. The grey box (top, separated by a dotted line) shows the distribution of total adaptation-relevant personnel (ARP) per team. Adaptation relevant personnel were defined as participants who performed three or more of the following roles. Note that this figure only

represents the participants in the FWC process (not all personnel that work at the city), and that we encouraged cross-department collaboration.

SI Table S2. Main variables, their definitions, and descriptive statistics pre- and post-intervention.

Variable	Variable Description	Pre-intervention			Post-intervention		
		n	M	SD	n	M	SD
**Actionable knowledge measure**							
Fit – "the way users perceive their information needs and their ability to deploy knowledge"[38]	Perception that the FWC tool supports decision-making, FWC information is applicable to their city's concern, FWC process fits how decisions are made, and FWC information can be applied to their context. (1=strongly disagree, 5=strongly agree)	200	3.90	.72	81	3.73	.73
Legitimacy – "perception that the production of information and technology has been respectful of stakeholders' divergent values and beliefs, unbiased in its conduct, and fair in its treatment of opposing views and interests"[38]	Perception that the FWC tool will be better accepted by their city than other tools and their city trusts projects informed by FWC information than other projects. (1=strongly disagree, 5=strongly agree)	199	3.35	.65	81	3.29	.75
Credibility – "scientific adequacy of the technical evidence and arguments"[33,38]	The extent of the perception that study partners including university researchers, the Climate Adaptation Partnership CAP/Regional Integrated and Assessments (RISAs), non-governmental organizations, and practitioners are credible. (1=not credible at all, 5=completely credible)	144	3.72	.69	62	3.75	.77
Interplay – "how new	The extent of the perception that the FWC	198	3.97	.62	80	3.88	.62

knowledge interacts with other types of knowledge and different decision contexts”[33,38]	tool offers new information, is different from other tools already used, and that the FWC tool will be used with other existing tools to support decision-making. (1=strongly disagree, 5=strongly agree)						
Usefulness – how useful knowledge is in decision making	The extent of the perception that the FWC tool identifies stormwater system components that are most at risk and capable of be adapted, finds their city-relevant climate and socioeconomic information, identifies communities in their city in most-need, supports communication, coordination, grant applications, identification of future investments, climate action plans, assist in adaptation implementation, and other. (1=not at all, 5=completely)	194	3.65	.73	80	3.42	.81
Adaptation intentions	Whether a list of 20 adaptation actions had been taken or were planned such as gathering more information about hazards or open space preservation. (0-20)	200	6.54	3.27	84	5.26	3.26
Adaptation-relevant personnel	Number of participants in a city reporting performing more than the sample mean (more than three tasks) number of adaptation-relevant tasks in their work, including implementing adaptation interventions, developing or updating climate action plans, guiding stormwater system investment, and other.	200	0.70	0.46	-	-	-

Note. See Methods section for complete description of variables including reliability scores. See Table S3 for more details about tests of proportion conducted for each intended adaptation action.

SI Table S3. Differences in individual adaptation action intentions, pre- and post-intervention.

Intention	Proportion selected		X^2	p-value
	Pre	Post		
**1: Gathering more information about hazards**	0.76	0.60	6.55	.011
2: Tidal barriers	0.16	0.12	0.50	.481
3: Natural or green infrastructure projects	0.45	0.57	3.02	.082
4: Building new levees or sea walls	0.18	0.11	1.84	.175
5: Installing backflow prevention devices (e.g., gates or check valves)	0.39	0.29	2.13	.145
6: Dry flood-proofing (e.g., watertight doors)	0.11	0.08	0.21	.644
7: Wet flood-proofing (e.g., submersible pump stations)	0.25	0.14	3.08	.079
8: Retreating from shorelines	0.06	0.08	0.21	.647
9: Relocation of assets	0.19	0.19	<0.001	.999
10: Open space preservation	0.44	0.39	0.28	.600
11: Urban forestry	0.16	0.11	0.76	.384
12: Low-impact development projects (e.g., grass swales, wet or dry ponds, green roofs, permeable pavements, etc.)	0.52	0.52	<0.001	.999
13: Installing larger pipes	0.52	0.38	3.74	.053
**14: Maintaining drainage ditches and pipes for higher run off**	0.76	0.61	6.05	.014
15: Raising stormwater inlets	0.15	0.07	2.32	.128
16: Installing pumps to force out water	0.27	0.23	0.59	.288
**17: Building additional conveyance or treatment capacity**	0.34	0.19	5.65	.017
**18: Installing detention or retention ponds to control runoff**	0.67	0.48	8.57	.003
19: Other stormwater retrofits not	0.13	0.06	2.04	.154

mentioned above				
20: Other measures not included above	0.08	0.05	0.34	.560

Note: Bold text denotes p<.05. These tests do not account for engagement group and no correction for multiple tests were performed; results should be interpreted with caution and should be treated as suggestive of patterns.

SI Table S4. Effects of intervention by engagement on adaptation intentions.

	IRR [95% C.I.]	Z	p	df
Time (post-intervention)	0.521 [0.353, 0.767]	-3.318	0.001	268
Engagement				
Web-assisted	0.811 [0.658, 0.999]	-1.976	0.049	268
In-Person	1.027 [0.825, 1.278]	0.237	0.813	268
Time × Web-assisted	1.116 [0.816, 1.525]	0.690	0.491	268
Time × In-Person	1.065 [0.774, 1.464]	0.389	0.698	268
State				
Florida	0.952 [0.703, 1.288]	-0.323	0.747	268
Louisiana	1.226 [0.873, 1.721]	1.181	0.239	268
Mississippi	0.752 [0.494, 1.144]	-1.338	0.182	268
Texas	1.113 [0.813, 1.523]	0.670	0.504	268
Adaptation relevant	1.141 [0.955, 1.363]	1.461	0.145	268
Time × Adaptation relevant	1.419 [1.004, 2.004]	1.993	0.047	268
Intercept	5.804 [4.120, 8.176]	10.101	<0.001	268
**Random effects**				
Community, σ^2_{u0}	0.030			
Participant, σ^2_{u1}	0.071			
Num. Observations	284			
Num. Imputations	20			

Notes. Incidence rate ratios (IRR) are reported to aid interpretation of effect sizes for Poisson models. Reference groups are self-guided engagement group and Alabama (state). Estimates are pooled from 20 multiply imputed datasets; all estimates represent pooled summaries. Community variance = 0.030 and participant variance = 0.071 across 20 imputations; there are no residual errors in Poisson multilevel models.

SI Table S5. Effects of intervention by engagement on fit.

	β [95% C.I.]	β*	Z	p	df
Time (post-intervention)	-0.705 [-1.213, -0.197]	-0.502	-2.731	0.007	257
<u>Engagement</u>					
Web-assisted	0.086 [-0.193, 0.366]	0.119	0.607	0.544	267
In-Person	-0.021 [-0.320, 0.278]	-0.029	-0.138	0.891	267
Time × Web-assisted	0.378 [-0.040, 0.796]	0.521	1.782	0.076	267
Time × In-Person	0.145 [-0.277, 0.567]	0.200	0.677	0.499	262
<u>State</u>					
Florida	-0.008 [-0.404, 0.387]	-0.011	-0.041	0.967	264
Louisiana	-0.096 [-0.544, 0.352]	-0.132	-0.422	0.673	264
Mississippi	0.100 [-0.429, 0.629]	0.138	0.373	0.709	264
Texas	-0.135 [-0.545, 0.276]	-0.185	-0.646	0.519	263
Adaptation relevant	0.289 [0.039, 0.539]	0.156	2.278	0.023	267
Time × Adaptation relevant	0.420 [-0.033, 0.874]	0.227	1.825	0.069	247
Intercept	3.666 [3.211, 4.121]	0.078	15.868	<0.001	264
**Random effects**					
Community, σ^2_{u0}	0.043 [0.040, 0.047]				
Participant, σ^2_{u1}	0.084 [0.071, 0.096]				
Residual, σ_e	0.618 [0.613, 0.629]				
Num. Observations	284				
Num. Imputations	20				

Notes. Standardized coefficients (β*) are reported to aid interpretation of effect sizes for linear models. Reference groups are self-guided engagement group and Alabama (state). Estimates are pooled from 20 multiply imputed datasets; all estimates represent pooled summaries.

SI Table S6. Effects of intervention by engagement on legitimacy.

	β [95% C.I.]	β*	Z	p	df
Time (post-intervention)	-0.422 [-0.922, 0.077]	-0.339	-1.665	0.097	234
<u>Engagement</u>					
Web-assisted	0.086 [-0.143, 0.315]	0.126	0.739	0.461	265
In-Person	0.090 [-0.158, 0.337]	0.131	0.713	0.477	267
Time × Web-assisted	0.290 [-0.085, 0.665]	0.426	1.525	0.128	262
Time × In-Person	0.246 [-0.136, 0.628]	0.361	1.270	0.205	243
<u>State</u>					
Florida	-0.213 [-0.515, 0.089]	-0.313	-1.390	0.166	266
Louisiana	-0.333 [-0.675, 0.009]	-0.489	-1.918	0.056	267
Mississippi	0.095 [-0.303, 0.494]	0.140	0.472	0.638	267
Texas	0.068 [-0.247, 0.383]	0.100	0.424	0.672	265
Adaptation relevant	0.038 [-0.203, 0.279]	0.022	0.311	0.756	266
Time × Adaptation relevant	0.236 [-0.205, 0.678]	0.136	1.054	0.293	236
Intercept	3.378 [3.006, 3.750]	0.113	17.873	<0.001	266
**Random effects**					
Community, σ^2_{u0}	0.000 [0.000, 0.000]				
Participant, σ^2_{u1}	0.091 [0.081, 0.105]				
Residual, σ_e	0.600 [0.593, 0.609]				
Num. Observations	284				
Num. Imputations	20				

Notes. Standardized coefficients (β*) are reported to aid interpretation of effect sizes for linear models. Reference groups are self-guided engagement group and Alabama (state). Estimates are pooled from 20 multiply imputed datasets; all estimates represent pooled summaries. Community level variance is estimated despite minimal clustering effects due to cluster randomized design.

SI Table S7. Effects of intervention by engagement on credibility.

	β [95% C.I.]	β*	Z	p	df
Time (post-intervention)	0.018 [-0.554, 0.589]	-0.027	0.062	0.951	87
<u>Engagement</u>					
Web-assisted	0.030 [-0.247, 0.307]	0.042	0.215	0.830	137
In-Person	0.064 [-0.224, 0.352]	0.090	0.439	0.661	178
Time × Web-assisted	-0.105 [-0.594, 0.384]	-0.148	-0.427	0.671	69
Time × In-Person	0.046 [-0.377, 0.469]	0.065	0.216	0.830	148
<u>State</u>					
Florida	0.120 [-0.244, 0.484]	0.170	0.650	0.517	179
Louisiana	0.164 [-0.240, 0.568]	0.231	0.798	0.426	212
Mississippi	-0.089 [-0.579, 0.402]	-0.124	-0.357	0.721	181
Texas	0.022 [-0.344, 0.387]	0.031	0.117	0.907	229
Adaptation relevant	0.217 [-0.069, 0.503]	0.120	1.504	0.135	115
Time × Adaptation relevant	-0.046 [-0.539, 0.447]	-0.026	-0.185	0.854	103
Intercept	3.440 [3.017, 3.862]	-0.145	16.053	<0.001	107
**Random effects**					
Community, σ^2_{u0}	0.002 [0.000, 0.019]				
Participant, σ^2_{u1}	0.219 [0.166, 0.279]				
Residual, σ_e	0.531 [0.485, 0.572]				
Num. Observations	284				
Num. Imputations	20				

Notes. Standardized coefficients (β*) are reported to aid interpretation of effect sizes for linear models. Reference groups are self-guided engagement group and Alabama (state). Estimates are pooled from 20 multiply imputed datasets; all estimates represent pooled summaries. Community level variance is estimated despite minimal clustering effects due to cluster randomized design.

SI Table S8. Effects of intervention by engagement on interplay.

	β [95% C.I.]	β*	Z	p	df
Time (post-intervention)	-0.531 [-0.943, -0.120]	-0.367	-2.543	0.012	242
<u>Engagement</u>					
Web-assisted	-0.046 [-0.286, 0.193]	-0.075	-0.382	0.703	266
In-Person	0.065 [-0.191, 0.322]	0.105	0.500	0.617	266
Time × Web-assisted	0.074 [-0.270, 0.418]	0.120	0.424	0.672	267
Time × In-Person	0.091 [-0.261, 0.442]	0.146	0.506	0.613	258
<u>State</u>					
Florida	-0.188 [-0.534, 0.158]	-0.303	-1.069	0.286	259
Louisiana	-0.343 [-0.736, 0.050]	-0.553	-1.717	0.087	260
Mississippi	-0.065 [-0.533, 0.402]	-0.105	-0.275	0.783	260
Texas	-0.042 [-0.403, 0.318]	-0.068	-0.232	0.817	258
Adaptation relevant	-0.033 [-0.244, 0.178]	-0.021	-0.310	0.757	264
Time × Adaptation relevant	0.375 [0.003, 0.748]	0.238	1.986	0.048	224
Intercept	4.135 [3.743, 4.527]	0.277	20.762	<0.001	261
**Random effects**					
Community, σ^2_{u0}	0.028 [0.026, 0.031]				
Participant, σ^2_{u1}	0.147 [0.131, 0.157]				
Residual, σ_e	0.458 [0.449, 0.471]				
Num. Observations	284				
Num. Imputations	20				

Notes. Standardized coefficients (β*) are reported to aid interpretation of effect sizes for linear models. Reference groups are self-guided engagement group and Alabama (state). Estimates are pooled from 20 multiply imputed datasets; all estimates represent pooled summaries.

SI Table S9. Effects of intervention by engagement on usefulness.

	β [95% C.I.]	β*	Z	p	df
Time (post-intervention)	-0.587 [-1.104, -0.070]	-0.174	-2.234	0.026	259
Engagement					
Web-assisted	0.226 [-0.046, 0.499]	0.294	1.635	0.103	256
In-Person	0.226 [-0.067, 0.520]	0.294	1.517	0.130	259
Time × Web-assisted	-0.037 [-0.453, 0.380]	-0.048	-0.173	0.863	263
Time × In-Person	0.129 [-0.292, 0.549]	0.167	0.603	0.547	260
State					
Florida	0.125 [-0.248, 0.498]	0.163	0.661	0.509	262
Louisiana	0.002 [-0.423, 0.427]	0.003	0.010	0.992	260
Mississippi	0.142 [-0.359, 0.642]	0.184	0.557	0.578	263
Texas	0.264 [-0.124, 0.653]	0.344	1.339	0.182	263
Adaptation relevant	0.049 [-0.218, 0.317]	0.025	0.363	0.717	261
Time × Adaptation relevant	0.559 [0.095, 1.023]	0.286	2.372	0.018	252
Intercept	3.302 [2.858, 3.747]	-0.296	14.628	<0.001	257
**Random effects**					
Community, σ^2_{u0}	0.011 [0.006, 0.017]				
Participant, σ^2_{u1}	0.210 [0.190, 0.236]				
Residual, σ_e	0.599 [0.587, 0.611]				
Num. Observations	284				
Num. Imputations	20				

Notes. Standardized coefficients (β*) are reported to aid interpretation of effect sizes for linear models. Reference groups are self-guided engagement group and Alabama (state). Estimates are pooled from 20 multiply imputed datasets; all estimates represent pooled summaries.

SI Table S10. Intended adaptation actions mediated by fit.

Predictor variables	Within pre-intervention B	SE	p	Pre-test predicting post-intervention B	SE	p
Direct effects						
Fit -> Adaptation intentions	-.163	.325	.617	.210	.370	.572
Engagement group -> Fit	-.010	.066	.877	-.010	.076	.894
Engagement group -> Adaptation intentions	.092	.301	.761	-.119	.343	.730
Indirect effects						
Engagement group -> Adaptation intentions	.002	.011	.883	-.002	.017	.903

Note. Estimates are pooled from 20 multiply imputed datasets; coefficients, standard errors/confidence intervals, and p-values represent pooled summaries.

SI Table S11. Intended adaptation actions mediated by legitimacy.

Predictor variables	Within pre-intervention B	SE	p	Pre-test predicting post-intervention B	SE	p
Direct effects						
Legitimacy -> Adaptation intentions	-.429	.415	.306	-.464	.356	.193
Engagement group -> Legitimacy	.064	.071	.370	.064	.060	.290
Engagement group -> Adaptation intentions	-.093	.351	.790	.123	.301	.684
Indirect effects						
Engagement group -> Adaptation intentions	-.027	.049	.582	-.029	.033	.373

Note. Estimates are pooled from 20 multiply imputed datasets; coefficients, standard errors/confidence intervals, and p-values represent pooled summaries.

SI Table S12. Intended adaptation actions mediated by credibility.

Predictor variables	Within pre-intervention B	SE	p	Pre-test predicting post-intervention B	SE	p
Direct effects						
Credibility -> Adaptation intentions	.007	.362	.985	.124	.425	.771
Engagement group -> Credibility	-.006	.069	.928	-.006	.082	.939
Engagement group -> Adaptation intentions	.093	.325	.776	-.119	.381	.755
Indirect effects						
Engagement group -> Adaptation intentions	.001	.012	.957	-.002	.021	.922

Note. Estimates are pooled from 20 multiply imputed datasets; coefficients, standard errors/confidence intervals, and p-values represent pooled summaries.

SI Table S13. Intended adaptation actions mediated by interplay.

Predictor variables	Within pre-intervention B	SE	p	Pre-test predicting post-intervention B	SE	p
Direct effects						
Interplay -> Adaptation intentions	-.540	.440	.225	.112	.377	.767
Engagement group -> Interplay	.023	.068	.729	.023	.057	.681
Engagement group -> Adaptation intentions	-.108	.352	.760	.090	.302	.765
Indirect effects						
Engagement group -> Adaptation intentions	-.013	.051	.800	.003	.013	.839

Note. Estimates are pooled from 20 multiply imputed datasets; coefficients, standard errors/confidence intervals, and p-values represent pooled summaries.

SI Table S14. Interview codebook.

Theme	Parent code	Child code	Definition (with hypotheticals)
Use	Use	Instrumental use	Examples of use such as to secure funding/grants, inform new projects, as communication tool, or plans to use tool in this way
		Symbolic use	Validation of existing perceptions, reinforcing system understanding, confirmation that adaptation plans already in pursuit are a good idea, results can be used to substantiate or argue for pre-existing plan to city council or public; "this is a really nice output that we can use to support the plan we've already put to city council for our stormwater system update"
		Conceptual use	Evidence of learning, changing perceptions, things that were surprising, thinking about a problem differently
	Non-use	Instrumental non-use	No use of tool, information in tool, or tool outputs to support actions for city
		Symbolic non-use	Rejection of information from tool because it did not validate existing knowledge, contradicts what participant knows from their own work and experiences
		Conceptual non-use	Pushback against information provided in tool, challenging conclusions from tool
Actionable knowledge indicators	Fit	High fit	Tool supports decision-making, data is applicable to community's concerns, the process aligns with how the participant makes decisions, participant feels that the climate profiles and/or neighborhood at risk data can be incorporated into their decision-making, the tool/data feels relevant to them, they think tool could be helpful for actions they perform
		No or low fit	Tool does not support decision-making, data is not applicable to community's concerns, the process does not align with how the participant or city makes decisions, participant feels that the climate profiles and/or neighborhood at risk data cannot be incorporated into their decision-making, the tool/data feels irrelevant to them
	Interplay	High interplay	Tool complements existing knowledge, information, tools used by the city team

	No or low interplay	Tool cannot be used in combination with other tools or other tools are prioritized
Credibility	High credibility	Participant expresses trust in researchers, tool, data
	No or low credibility	Participant expresses skepticism about researchers, tool, data
Legitimacy	High legitimacy	Participant feels that decisions informed by tool will be better accepted or more trusted by the community, including the public, elected officials, colleagues; Process by which tool was created/co-produced or engagement is run is transparent and makes clear who or what was involved
	No or low legitimacy	Participant feels that decisions informed by the tool will be rejected or less trusted by community, or participant feels the tool has no effect on how the community considers outcomes of its use; Process by which tool was created/co-produced or engagement is run is not transparent and does not make clear who or what was involved

Interview protocol

Online Interview Script

Introductory Script

Hi, my name is _________. I am a __________ at __________. I would like to talk to you about your experience working on the FloodWise Communities vulnerability assessment over the past few months. Our goal is to take what we've learned from this project to support other cities in the region and across the country to do similar work. Your feedback about what worked and what didn't will be very helpful as we reflect on how to scale up the project, so thank you for making the time.

The interview should take no more than an hour. We will not collect any personal information from you, and your responses will remain anonymous. Participation in this study is completely voluntary. Even if you decide to participate, you may change your mind and stop at any time. You may choose not to answer questions, or to stop participating in the interview for any reason.

With your consent, we would like to record this interview. The recording will be destroyed once the interview is transcribed. *Do you give consent to be recorded: yes OR no?*

Thank you.

*****start recording*****

- **Personal Information:** Can you tell me about your role in your [Place]?
  - **[Probe]** And your experience with stormwater systems or other natural hazards?
  - **[Probe]** How long have you worked in this role?

- **Program Expectations:**
  - **Program draw:** Why did you, personally, participate in FloodWise Communities?
    - **[Probe]** Who brought you to the project? Why did they want you to be on the team?
    - **[Probe] Program information:** How did you learn about FloodWise Communities?
  - **Program expectations**: What did you hope that the program would offer you, at the beginning of engagement?
  - **Program reservations:** Was there anything that you were concerned about in regards to participating in the program?
  - **Possible engagement prep:** Looking back, is there anything you wish you had known about the program ahead of time?

      - **[Probe]** Is there anything you wish FloodWise Communities had communicated to you?
- **Recruitment:**
  - **Intersectional Recruitment**: Do you think your FloodWise Project Team made an effort to include people from different departments and external organizations?
      - **[Probe: If made effort, and others joined]** What was the impact of involving people from different departments and external organizations?
  - **Intersectional Perceived Value:** Are there any departments or external organizations you wish would have participated in the engagement, but didn't?
      - **[Probe]** Why do you wish they had participated?
      - **[Probe]** Why do you think they did not participate?
  - **[only for self-guided engagements] Pre-engagement work** What were the challenges to get your team set up to start the assessment?

TRANSITION: Now we're going to talk about your actual experience in the program.

- **Program Assessment:**
  - **General impression of value**: How valuable was participating in this program? And when I say "valuable", that can mean anything that comes to your mind. (**Note: Some of the following questions may be answered in this question, so adjust which ones you ask based on their response to this first question)**
      - [**Probe**] Why was it valuable/not valuable?
      - **[May be redundant with answers above; use discretion] Applicability to the local context**: How do you think you are going to use what you learn in the program in [village/city/county/parish name]?
          - How do you think you will use the different program outputs in [village/city/county/parish name]? (e.g. Climate Profile, Vulnerability assessment, Neighborhoods at Risk)
  - **[only for in-person and online engagements] Post-engagement work:** Did you keep working on your assessment after the workshop? How was that process for you?
  - **Co-production aspect:** What do you know about how the FloodWise Communities tool was created?
  - **[If they don't know/remember from engagement or post-survey]** The FloodWise Communities tool was developed by practitioners and scientists in 5 Great Lakes cities to address stormwater concerns.
  - Was this apparent in your engagement at all? [For example: was this part of the reason you chose to join the engagement, did the tools seem to be a better or worse fit for your decision-making context, were the types of data in the engagement relevant, etc.]

  - **Assessment of level of engagement and support:** How do you feel about the level of support that you received from our team while completing the assessment using the Floodwise Communities tool?
      - **Support for each of the elements**: Think about your experiences working with the following elements (Vulnerability assessment, Climate Profile, Flood Factor, and Neighborhoods at Risk). How easy was it to use/navigate each of them?

        - [Probe for any elements that they forget to talk about]
    - Relevance of information: Did you find the information in the Climate Profile and Neighborhoods at Risk relevant to your work? Why or why not?
    - **Credibility of information providers:** What do you know about the sources of the information in the Climate Profile and Neighborhoods at Risk?
        - Did you find their information credible? Why or why not?
    - [If they do not know/remember data sources]To refresh your memory, most of the data from the climate models in the Climate Profile was provided by the National Center for Atmospheric Research in Boulder, CO, the Southern Regional Climate Center at Texas A&M, and NOAA, and the information in Neighborhoods at Risk was a combination of census data and FEMA data
        - Now that you know who this information came from, do you feel differently about its credibility?
        - Do you feel differently about its relevance?
    - **Legitimacy of information providers:** Do you feel like GLISA, SCIPP, Adaptation International and Headwaters Economics were qualified to help your community with its stormwater management? Why or why not?
        - **[For in person and webinar]** Do you feel like your facilitators, specifically, were qualified to help your community with stormwater management? Why or why not?
    - **Contextual factors**: Did any factors {e.g. recent weather event, covid-related delays, losing team members, etc. } make it more challenging to complete the vulnerability assessment?
        - Did any factors make it easier to complete the vulnerability assessment?
    - **[Probe:] External help:** Did you consult with anyone outside the project team to complete your vulnerability assessment?
        - [If yes] Could you tell us who you consulted and the reason why?

    - **Engagement mode appeal:** Would you have participated in the program if the only options were for a [the two options that were not assigned for this city. E.g. self-guided/webinar/in person] workshop? Why or why not?
    - **Networks/learning**: Do you or your colleagues intend to share what you learned in this experience with anyone else? Why/why not?
        - [If yes] With whom? Why?
    - **Suggestions for improvement**: If it were up to you, is there anything you would have changed about the vulnerability assessment?
        - [Same questions as above, for Neighborhoods at Risk and Climate Profile]

Now we'll be pivoting to discuss questions relating to your [city/county/parish/village]'s disaster experience and adaptive capacity.

Adaptive Capacity

- **Disaster experience informing perception (maybe refer back to engagement answers)**: Could you describe any previous disasters that impacted [village/city/county/parish name] and how they prepared you for future natural hazards?

- **Previous adaptation actions:**
    - **Previous adaptation actions**: Before participating in the program, did you undertake any adaptation actions relating to stormwater management? [Give examples if they cannot come with any. e.g. built a seawall, redesigned pipelines, outreach educational programs]
        - [If no]: What specific types of resources might enable you to undertake adaptation actions? [Refer examples they used in the workshop - funds/personnel/administration/data/engineering and infrastructure]
        - [If yes] Could you walk us through the process [keep for each action]?
            - **[Probe: MOTIVATION]:** What prompted you to undertake [insert action here]?
            - **[Probe: RESOURCES]** What specific types of resources (e.g. funds/personnel/administration/data/engineering and infrastructure) enabled you to undertake [insert action here]?
            - **[Probe: CHALLENGES]**: What were major challenges that you encountered when implementing [insert action here]? How were these overcome?

- **AC perception and future actions:**
    - **Priority changes**: How has participating in this program affected your sense of what your priorities are in relation to natural hazard adaptation?

    - **Impact of the program on future efforts**:
        - How has participating in the FloodWise vulnerability assessment changed your view about what communities need to adapt to future natural hazards?
        - After participating in the program, do you think you are better prepared for future stormwater management? How so?
        - (**May have covered this in "Priority changes" question**): After participating in the program, have you planned any new action to deal with future stormwater management issues? What are they?
            - **[Probe if yes] Responses from others**:
            - Do you think decision-makers in your [city/county/parish/town] would be supportive of future efforts to deal with stormwater? [Based on the concrete examples given by the respondee]
            - How will residents in your [city/county/parish/town name] respond to future efforts to deal with stormwater?
    - **Scalability/Success in the implementation:** From your experience, what do you think might be needed for encouraging adaptive action in **smaller or mid-sized** jurisdictions (county, city, parish) in your region?

Now we'll be pivoting to discuss questions relating to your [city/county/parish/village]'s social vulnerability and equity.

Vulnerability and Equity

- **Action Informed:** Is participating in the FloodWise Communities program going to inform equity or social vulnerability choices in your [village/town/city/parish] in the future adaptation actions? How?

- **Intention shift:** Based on your experience in the program, has your intention to include social vulnerability or equity in climate adaptation actions changed? Why? How?
    - **Challenges:** Are there any major challenges you anticipate will come up when implementing these social vulnerability actions?
- **Vulnerability and equity perceptions:** How do you define social vulnerability to natural hazards?
    - **[Probe]:** How do you think the term applies to your community?
- **Equity**: How do you define equity in relation to natural hazards?
    - **[Probe]:** How do you think the term applies to your community?
- **Equity and Social Vulnerability:** In your opinion, how does social vulnerability relate to equity in terms of natural hazards?
- **Prior Social Vulnerability Actions:** Before participating in the program, did any of your work include efforts to address social vulnerability or equity?
    - [Probe if yes] Could you describe these efforts?
    - [Probe if they do have efforts to describe] Can you describe any challenges you or your colleagues faced when designing or implementing these efforts?
- **Social Vulnerability Shift**: Based on your experience in the program, did your perception or understanding of social vulnerability change? How so?
    - [Probe]: For example, did participation help to identify places that are more at risk because of their social characteristics? if yes please explain?
- **Equity shift:** Has your understanding of equity related to natural hazards changed after participating in FloodWise Communities? How?

Now we're going to wrap up with a few closing questions about your overall experience in the program.

- **Suggested changes** [For webinar and in-person]: Is there anything you would change about the engagement process? [For self-guided]: is there anything you would change about the tutorial elements?

- **Potential for post-engagement support:** What additional technical resources or support that we did not offer would help you complete your vulnerability assessment?

- **Wrap-up:** Is there anything else you would like to share?

Thanks for taking the time to speak with me, if you have any additional thoughts or questions, please feel free to email them to me, or we can schedule a follow up call at your convenience.
